# High-density diffuse optical tomography for imaging human brain function

**DOI:** 10.1063/1.5086809

**Published:** 2019-05-22

**Authors:** Muriah D. Wheelock, Joseph P. Culver, Adam T. Eggebrecht

**Affiliations:** 1 Mallinckrodt Institute of Radiology, Washington University School of Medicine, St. Louis, Missouri 63110, USA; 2 Department of Physics, Washington University in St. Louis, St. Louis, Missouri 63130, USA; 3 Department of Biomedical Engineering, Washington University in St. Louis, St. Louis, Missouri 63130, USA; 4 Division of Biology and Biomedical Sciences, Washington University in St. Louis, St. Louis, Missouri 63130, USA

## Abstract

This review describes the unique opportunities and challenges for noninvasive optical mapping of human brain function. Diffuse optical methods offer safe, portable, and radiation free alternatives to traditional technologies like positron emission tomography or functional magnetic resonance imaging (fMRI). Recent developments in high-density diffuse optical tomography (HD-DOT) have demonstrated capabilities for mapping human cortical brain function over an extended field of view with image quality approaching that of fMRI. In this review, we cover fundamental principles of the diffusion of near infrared light in biological tissue. We discuss the challenges involved in the HD-DOT system design and implementation that must be overcome to acquire the signal-to-noise necessary to measure and locate brain function at the depth of the cortex. We discuss strategies for validation of the sensitivity, specificity, and reliability of HD-DOT acquired maps of cortical brain function. We then provide a brief overview of some clinical applications of HD-DOT. Though diffuse optical measurements of neurophysiology have existed for several decades, tremendous opportunity remains to advance optical imaging of brain function to address a crucial niche in basic and clinical neuroscience: that of bedside and minimally constrained high fidelity imaging of brain function.

## I. INTRODUCTION: HEMODYNAMIC IMAGING OF HUMAN BRAIN FUNCTION

Imaging spatially and temporally distributed brain activity has revolutionized our understanding of the brain.^1–4^ The interacting brain systems supporting our thoughts and actions—from sensing the visual world, to communicating, to maintaining attention and control, to daydreaming or sleeping—are accessible to quantitative investigation through functional imaging techniques.^4–6^ Additionally, functional brain imaging has provided insight into neurological and psychiatric disorders such as Alzheimer’s disease,^7^ autism spectrum disorder (ASD),^8–10^ and stroke.^11,12^ However, optimizing neuroimaging technologies as tools for understanding these disorders and tracking their progression presents significant challenges. Optical neuroimaging techniques offer a unique opportunity for safe, wearable, and portable methods for measuring brain function at the clinical bedside and in naturalistic settings. This review will discuss recent advancements in high-density diffuse optical tomography (HD-DOT) methods that have led to improved image quality and reliability in noninvasive optical mapping of human brain function.

**FIG. 1.**
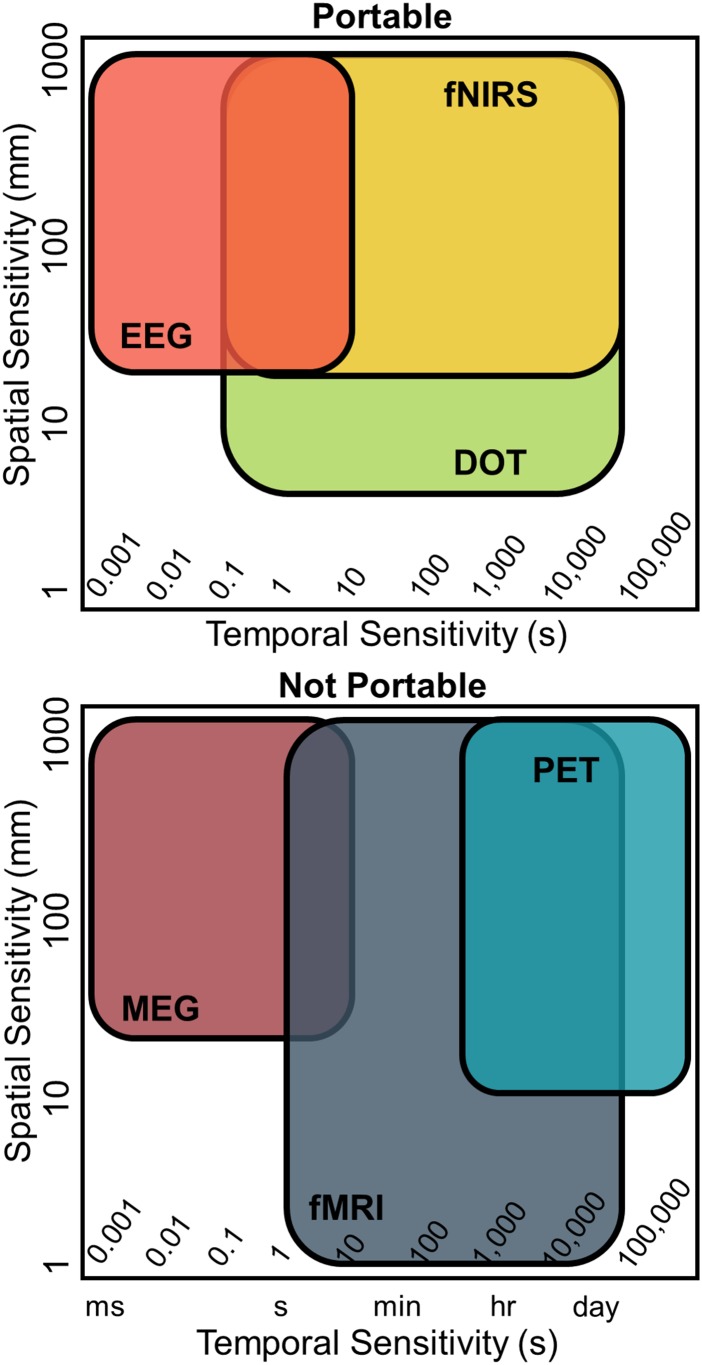
Spatial, temporal, and mobility domains of the leading methods available for measuring human brain function. Each colored region represents a rough estimate of the spatial and temporal capabilities for each modality. EEG, fNIRS, and DOT systems can be deployed at the bedside, in the laboratory, or in the hospital. MEG, fMRI, and PET machines require dedicated facilities, are immobile, and patients/participants must be transported to the facilities for imaging. EEG, electroencephalography; fNIRS, functional near infrared spectroscopy; DOT, Diffuse Optical Tomography; MEG, magnetoencephalography; PET, positron emission tomography; and fMRI, functional magnetic resonance imaging.

The diverse set of physiological dynamics encompassing neurological processing engenders multiple opportunities for measurements of human brain function across a remarkably wide range of spatial and temporal scales (Fig. 1). When a part of the brain is active, the local firing of neurons gives rise to varying electrical field potentials that can be measured at the millisecond scale invasively with electrocorticography (ECoG) or noninvasively with electro/magneto encephalography (EEG/MEG). This local firing of neurons triggers a complex neurovascular cascade^13–15^ that produces a dramatic increase in glucose use and local blood flow resulting in a large increase in oxygen availability.^16,17^ The dynamic changes in glucose metabolism and blood flow can be measured by positron emission tomography (PET). The resulting relative changes in local concentrations of oxygenated (HbO_2_), deoxygenated (HbR), and total hemoglobin (HbT) give rise to a blood oxygenation level dependent (BOLD) signal as measured by functional magnetic resonance imaging (fMRI)^18–23^ and, differently, by functional near infrared spectroscopy (fNIRS)^24^—the basis for HD-DOT. Each of these measurement methods differs in their practical strengths and limitations (Table I). For example, PET utilizes ionizing radiation that is generally prohibited for research use in children. The strong electromagnetic fields required for fMRI are unsafe for participants with implanted active electronic devices (e.g., pacemakers, deep brain stimulators, and cochlear implants). The wearable, portable nature of optical technologies opens the door to bedside and minimally constrained imaging of functional brain health,^25–28^ in settings more ecologically natural than MRI.^24,29–33^ Given these strengths, fNIRS technologies are uniquely suited to studies involving infants and toddlers,^25,34–39^ and they are ideal for use in clinical settings in which standards of clinical care lead to complex or untenable logistics for moving the patient to an MRI machine (e.g., if the patient is on a ventilator).

**TABLE I.** Comparison of functional neuroimaging techniques for applications in human subjects. HD-DOT, high density diffuse optical tomography; fNIRS, functional near infrared spectroscopy; DCS, diffuse correlation spectroscopy; fMRI, functional magnetic resonance imaging; PET, positron emission tomography; EEG, electroencephalography; ECoG, electrocorticography; MEG, magnetoencephalography. Boldface text highlights strong positive attributes while italicized text denotes negative attributes.

Contrast mechanism	Hemodynamics					Electromagnetic potentials		
Measurement technique	HD-DOT	fNIRS	DCS	fMRI	PET	EEG	ECoG	MEG
Spatial resolution	Med	*Low*	*Low*	**High**	Med	*Low*	**High**	Med
Temporal resolution	Med	Med	Med	Med	*Low*	**High**	**High**	**High**
Field-of-view	Med	Med	*Low*	**High**	**High**	Med	Med	Med
Nonionizing	**Yes**	**Yes**	**Yes**	**Yes**	*No*	**Yes**	**Yes**	**Yes**
Free of contraindications	**Yes**	**Yes**	**Yes**	*No*	*No*	**Yes**	**Yes**	**Yes**
Noninvasive	**Yes**	**Yes**	**Yes**	**Yes**	**Yes**	**Yes**	*No*	**Yes**
Wearable	**Yes**	**Yes**	**Yes**	*No*	*No*	**Yes**	Med	*No*
Naturalistic environment	**Yes**	**Yes**	**Yes**	*No*	*No*	**Yes**	*No*	*No*
Portable	**Yes**	**Yes**	**Yes**	*No*	*No*	**Yes**	*No*	*No*
Motion sensitivity	Med	Med	*High*	*High*	*High*	*High*	*High*	*High*
Cost	Med	Med	Med	*High*	*High*	Med	Med	*High*

**FIG. 2.**
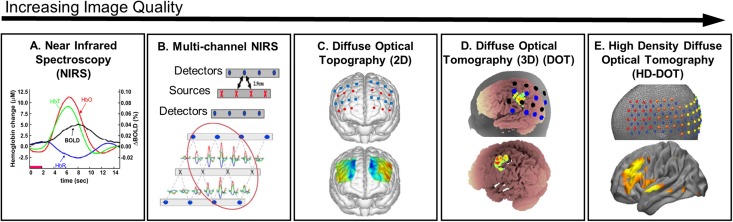
Image quality obtainable with fNIRS methods varies with the number and spatial overlap of the measurements. (a) A single source-detector pair measurement illustrates the similar time course of oxygenated hemoglobin (HbO), deoxygenated hemoglobin (HbR), and total hemoglobin (HbT) observed with fNIRS and that of the blood oxygen dependent (BOLD) signal observed using fMRI. [Reproduced with permission from D. A. Boas, A. M. Dale, and M. A. Franceschini, NeuroImage **23**(Suppl 1), S275–S288 (2004). Copyright 2004 Elsevier.] (b) Multichannel fNIRS utilizes measurements between several source-detector pairs to measure temporal hemodynamics across an expanded field of view of the brain. [Reproduced with permission from Huppert *et al.*, NeuroImage **29**, 368–382 (2006). Copyright 2006 Elsevier.] (c) Diffuse optical topography methods reconstruct multichannel fNIRS measurements into two-dimensional (2D) maps with a spatial resolution of 1–2 cm. [Reproduced with permission from Yennu *et al.*, Sci. Rep. **6**, 30157 (2016). Copyright 2016 Springer Nature Publishing.] (d) Diffuse optical tomography (DOT) methods reconstruct spatially overlapping multidistance source-detector measurement channels into three-dimensional (3D) maps with some level of depth profiling. [Reproduced with permission from Custo *et al.*, NeuroImage **49**, 561–567 (2010). Copyright 2010 Elsevier.] (e) High density DOT (HD-DOT) methods use a nearest source-detector spacing of at most 15 mm to reconstruct 3D maps that have been shown to approach a spatial resolution comparable to that of fMRI. [Reproduced with permission from Hassanpour *et al.*, NeuroImage **117**, 319–326 (2015). Copyright 2015 Elsevier.]

Though fNIRS methods are deployable at the bedside, anatomical specificity is less precise and spatial resolution of the acquired images is lower than what is obtainable with fMRI (Fig. 1). Each single fNIRS measurement obtained from a given source-detector (SD) measurement pair recovers information about the underlying hemodynamics along a broad spatial path—including brain and superficial tissues—traversed by photons traveling from the source to the detector^24^ [Fig. 2(a)]. Acquiring data from multiple SD measurement pairs provides access to more hemodynamics even without utilizing imaging techniques [Fig. 2(b)].^40–42^ Diffuse optical topography techniques can reconstruct sparse multichannel fNIRS data into spatial maps with moderate spatial resolution but no depth information^43,230,231^ [Fig. 2(c)]. To improve the image quality of sparse fNIRS, spatially overlapping fNIRS measurements can be tomographically reconstructed to produce three-dimensional maps of brain function [Fig. 2(d)], a technique known as diffuse optical tomography (DOT).^31,44,57^ To further improve image quality, HD-DOT systems use a dense regular array of sources and detectors to obtain overlapping measurements at multiple distances. Herein, high-density is defined as a regular array, typically an interlaced lattice of sources and detectors, with a closest (a.k.a., nearest neighbor) SD distance of at most 15 mm^45^ [Fig. 2(e)]. This maximum distance of 15 mm for the nearest neighbor SD separation makes possible access to multiple SD distances, including out to 40 mm and beyond, that together provide measurements crucial for obtaining spatial maps of brain function comparable to fMRI. Indeed, advances in image quality obtained with HD-DOT, including a spatial resolution approaching that of fMRI,^25,46^ have been demonstrated in recovered maps of brain function using both task-based^25,33,45–59^ and resting state functional connectivity techniques.^25,26,46,60^

In this review, to contextualize challenges in HD-DOT system design, we will briefly describe the physical mechanisms underlying fNIRS measurements, and the theory underlying modeling of light propagation in tissue. We will then focus on optical-electronic instrumentation and cap design utilized in HD-DOT systems. We additionally highlight several validation studies of HD-DOT mapping of cortical activity and connectivity in response to tasks and during a resting state. We then discuss the use of HD-DOT in clinically oriented applications. Finally, we will briefly consider opportunities to further improve image quality, anatomical specificity, and reliability so that HD-DOT methods can realize their true potential in unconstrained and noninvasive assessment of human brain function in the clinic, in naturalistic and even remote settings, and in sensitive populations.

## II. OPTICAL IMAGING OF BRAIN FUNCTION: THEORETICAL BACKGROUND

### A. Photon diffusion through biological tissue

**FIG. 3.**
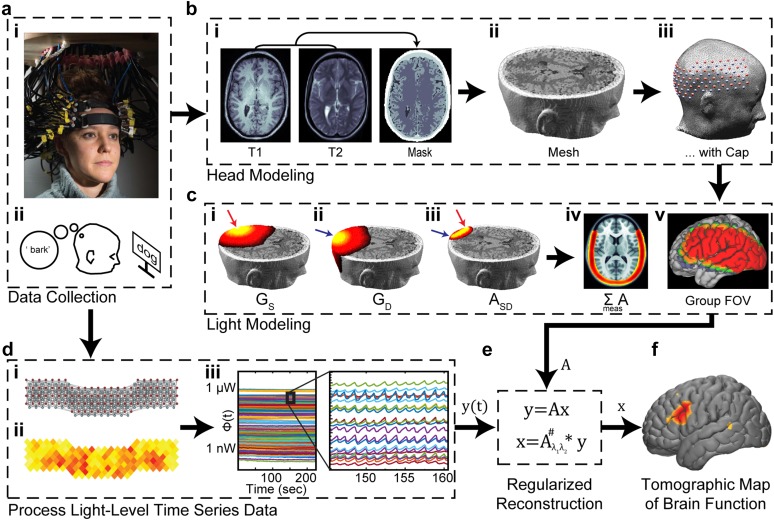
HD-DOT methods overview. (a) Data collection involves (i) locating source and detector positions on the head and (ii) recording light levels from the head of a participant. In this example, a stimulus paradigm involves the participant generating novel verbs in response to nouns presented on a monitor. (b) A head model for a given participant is created by (i) generating a subject-specific or atlas-based volumetric segmentation of the head tissue, (ii) building a high-density mesh, and (iii) placing the sources and detectors on the head mesh surface. (c) Using the head model, the sensitivity profile for (i) each source (G_s_), and (ii) each detector (G_d_) are calculated and (iii) combined into a sensitivity profile for each source-detector measurement pair A_SD_. (iv) The full system sensitivity ΣA can be visualized by summing the sensitivity of each measurement pair. (v) The modeled sensitivity can then be spatially registered to an atlas space for group-level analyses. (d) Separately, the collected light-level data are assessed for (i) noise and (ii) signal level quality, (iii) with high quality optical data clearly showing a pulse waveform. (e) After preprocessing, the optical data are combined with a regularized inverse of the sensitivity model to generate (f) anatomically-registered maps of cerebral hemodynamics reflecting brain function. [Adapted with permission from Eggebrecht *et al.*, Nat. Photonics **8**, 448–454 (2014). Copyright 2014 Springer Nature Publishing.]

**FIG. 4.**
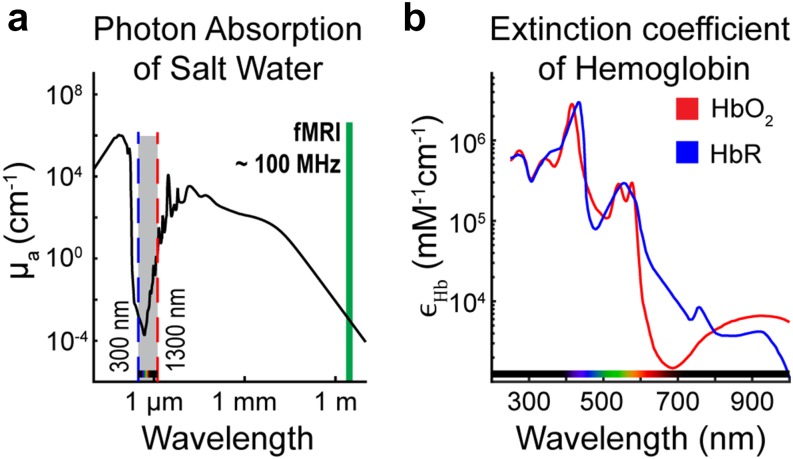
The optical window in biological tissue. (a) The absorption coefficient of salt water, a primary component of biological tissue, is low within the visible and near infrared (NIR) ranges of the electromagnetic spectrum (gray shading), thereby allowing photons within this range to penetrate multiple centimeters in tissue. The green line represents the frequencies of electromagnetic radiation typically used in functional MRI. (b) Utilizing multiple wavelengths within the visible and near infrared range enables spectroscopic unmixing of oxygenated (HbO_2_) and deoxygenated hemoglobin (HbR) chromophores due to their varying extinction coefficients.

In Secs. II B–VI, we will discuss how, with a high density array of sources and detectors and an appropriate model for light propagation, it is possible to accurately reconstruct brain function within the tissue volume from a set of these measurements collected on the surface (Fig. 3). The fundamental unit of an fNIRS measurement is a paired source and detector of near-infrared (NIR) light. In the late 1970’s, Jöbsis observed a range of wavelengths (∼700–1300 nm) in the electromagnetic spectrum wherein photons penetrate multiple centimeters through biological tissue^61^ and can provide direct measurements of hemodynamic physiology deep (>1 cm) in living intact tissue. The deeper penetration occurs within this “optical window” due to relatively weak absorbance of photons by the primary chromophores in biological tissue (water, lipids, and hemoglobin)^62^ (Fig. 4). Importantly, as will be discussed below, though the photon absorption is low, the scattering of photons is high in biological tissue and can be well approximated as a diffusive process.^63–67^ The local transient changes in local concentrations in HbO_2,_ HbR, and HbT brought about by varying brain activity are reflected in variance in the light levels of a given fNIRS SD measurement pair. Sections II B–II E discuss how to localize the changes within the volume from an HD set of measurements on the surface.

### B. Forward light modeling

In optical functional neuroimaging, the goal is to model how variations in light level measurements on the surface correspond to transient changes in optical properties within the volume. This relationship can be concisely described by

y=Ax, (1)

where ***y*** is a vector of measurements from the set of source-detector pairs (what we have), ***x*** represents the change in absorption and/or scattering at each point in the volume (what we want to know), and ***A*** is called the sensitivity matrix (also called the Jacobian) that relates differential changes in light measurements to differential changes in internal optical properties. This sensitivity matrix is constructed from a model, termed the forward light model, derived fundamentally from the Boltzmann Transport Equation (BTE), or, equivalently in this context, the Radiative Transport Equation (RTE). The BTE is a conservation equation that can be utilized to describe the flow of light energy *E* through a scattering medium (e.g., a head). This formalism is equivalent to a description of the flow of photons as $E=\frac{nhc}{\lambda }$, where *n* is the number of photons, *h* is Planck’s constant, *c* is the speed of light in a vacuum, and *λ* is the photon wavelength (see Table II for a list of the primary quantities discussed in this review with their units and typical values).

**TABLE II.** Constants and parameters for diffuse light transport.

			Value/typical value
			in biological tissue
Quantity	Symbol	Units	for λ = 850 nm
Radiance	I(**r**, t, s)	W/(cm^2^·sr)	n/a
Planck’s constant	h	J·s	6.626 × 10^−34^
Speed of light in a vacuum	c	m/s	2.998 × 10^8^
Index of refraction	n	Dimensionless	1.4
Speed of light in biological tissue	v = c/n	cm/ns	21.4
Absorption coefficient	μ_a_	cm^−1^	0.2 (gray matter^a^)
Scattering coefficient	μ_s_	cm^−1^	67 (gray matter^a^)
Anisotropy of scattering factor	g	Dimensionless	0.9
Reduced scattering coefficient	μ_s_′ = (1 − g)μ_s_	cm^−1^	6.7
Transport mean free path	L = 1/(μ_a_ + μ_s_′)	cm	0.14
Fluence rate	Φ(**r**, t)	W/cm^2^	n/a
Photon diffusion coefficient	D = v/3(μ_a_ + μ_s_′)	cm^2^/ns	1.03

^a^ Reference 75.

**FIG. 5.**
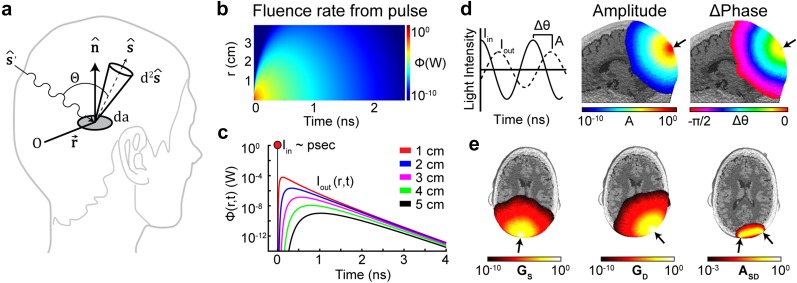
Modeling photon diffusion through biological tissue. (a) Schematic detailing relevant quantities for the RTE. See the text for details. (b) In response to a brief (∼ps) pulse of light deposited within an infinite homogeneous medium, the fluence rate behaves like a spherically decaying Gaussian in space and exponential in time. (c) The fluence rate response to the pulse in (b) evaluated at five distances away from the pulse deposition. Each line corresponds to data taken from a horizontal section of (b) and highlights the temporal broadening of the initial pulse. (d) In response to a ∼100 MHz intensity modulated light source on the surface of the head (black arrow), measurements of both amplitude (A) and phase shift (∆θ) can inform estimates of underlying optical properties. (e) The Green’s function of a continuous wave source (G_S_) on the surface (black arrow on left) and a detector (G_D_) some distance away (center arrow) are multiplied together at every location in space to derive the spatial distribution of the source-detector measurement sensitivity (A_SD_ right).

To construct the model, let us start by defining the energy radiance $I\left(\overset{⇀}{r},t,ŝ\right)$ (i.e., the energy flowing per unit time through an area per solid angle, in units of $\frac{\text{W}}{\text{c}{\text{m}}^{2}\text{ sr}}$)^68^ such that the differential energy *dE* flowing in a unit solid angle $d^{2}ŝ$ through an elemental area *da* with associated normal $\hat{n}$, at position $\overset{⇀}{r}$, in time *dt* is [Fig. 5(a)]

$$dE=I\left(\overset{⇀}{r},t,ŝ\right)ŝ\cdot \hat{n}da\;d^{2}ŝdt.$$ (2)

Here, we are simplifying by considering energy at a specific wavelength of light (e.g., *λ* = 850 nm) as opposed to a range of wavelengths, by assuming the scattering is elastic (Mie scattering dominates in biological tissue^69^), and by neglecting polarization, coherence, and nonlinearities. The radiance is proportional to the square of the electric field at position $\overset{⇀}{r}$, traveling in direction ŝ in time *dt*. The RTE states that in each infinitesimal element of the volume of the medium [Fig. 5(a)],

$$\begin{array}{ccc} \frac{1}{v}\frac{\partial I\left(\overset{⇀}{r},t,ŝ\right)}{\partial t} & = & \mu_{s}\int_{4\pi }f\left(ŝ,{ŝ}^{\prime }\right)I\left(\overset{⇀}{r},t,{ŝ}^{\prime }\right)d^{2}{ŝ}^{\prime }+q\left(\overset{⇀}{r},t,ŝ\right)\\ & & -\;ŝ\cdot \nabla I\left(\overset{⇀}{r},t,ŝ\right)-\left(\mu_{a}+\mu_{s}\right)I\left(\overset{⇀}{r},t,ŝ\right), \end{array}$$ (3)

where *v* is the speed of light in the medium ($v=\frac{c}{n}\approx 21.4\frac{\text{cm}}{\text{ns}}$, where *n* = 1.4 is the index of refraction in the medium); *μ*_*s*_ is the scattering coefficient (in units of $\frac{1}{\text{cm}}$); $f\left(ŝ,{ŝ}^{\prime }\right)$ is the scattering phase function, which is essentially the probability density of a photon scattering from direction ${ŝ}^{\prime }$ into direction ŝ; $q\left(\overset{⇀}{r},t,ŝ\right)$ is a source term (with units of $\frac{\text{W}}{\text{c}{\text{m}}^{3}\text{ sr}}$) representing power per volume emitted by sources at position $\overset{⇀}{r}$ in time *dt* in direction ŝ; and *μ*_*a*_ is the absorption coefficient of the medium (in units of $\frac{1}{\text{cm}}$). Conceptually, Eq. (3) states that the change in radiance (i.e., the change in optical power through a differential area and unit solid angle) at time *t* in direction ŝ at position $\overset{⇀}{r}$ is due to four possible quantities: (i) gains and losses in energy due to photons being scattered into direction ŝ and position $\overset{⇀}{r}$, (ii) gains in energy due to local sources of photons, (iii) changes in net energy flow into the differential volume, and (iv) losses in energy due to absorption and scattering, respectively. The absorption and scattering coefficients of the medium (e.g., scalp, skull, brain tissue, etc.) are wavelength dependent [*μ*_*a*_(*λ*), *μ*_*s*_(*λ*), respectively] and correspond to the reciprocal of the mean distance traveled by a photon before it is absorbed or scattered, respectively. More exactly, these coefficients represent the reciprocal of the mean distance traveled before a photon is absorbed/scattered in the absence of scattering/absorption. These distances are distinct from (and much smaller than) the transport mean-free-path (a.k.a., the random walk step) $l=\frac{1}{\mu_{a}+\mu_{s}^{\prime }}$, which represents the typical distance a collection of photons travels in a given medium before their directions effectively become randomized and uniformly distributed (i.e., isotropic). The reduced scattering coefficient $\mu_{s}^{\prime }$ includes information about the anisotropic scattering characteristic of the medium and will be mathematically derived below. To simplify, here we are treating the medium as if the index of refraction and the coefficients of absorption and scattering are constant throughout. We will deal with spatially and temporally variant optical properties below.

If we make the assumption that the radiance $I\left(\overset{⇀}{r},t,ŝ\right)$ is nearly isotropic to first order (i.e., uniform in all directions), then Eq. (3) can be simplified by expanding $I\left(\overset{⇀}{r},t,ŝ\right)$ into spherical harmonics and truncating after the first term (this is also referred to as the P_1_ approximation)^70–73,113^

$$I\left(\overset{⇀}{r},t,ŝ\right)=\frac{1}{4\pi }\Phi \left(\overset{⇀}{r},t\right)+\frac{3}{4\pi }ŝ\cdot J\left(\overset{⇀}{r},t\right),$$ (4)

where $\Phi \left(\overset{⇀}{r},t\right)$ is the fluence rate (a scalar intensity in units of $\frac{\text{W}}{\text{c}{\text{m}}^{2}}$), defined as the total power per area radiating radially outward from a volume element at position $\overset{⇀}{r}$ and time *t*

$$\Phi \left(\overset{⇀}{r},t\right)=\int_{4\pi }I\left(\overset{⇀}{r},t,ŝ\right)d^{2}ŝ,$$ (5)

and $J\left(\overset{⇀}{r},t\right)$ is the photon current flux (a vector intensity in units of $\frac{\text{W}}{\text{c}{\text{m}}^{2}}$), essentially the directed vector sum of the radiance emerging from the volume element

$$J\left(\overset{⇀}{r},t\right)=\int_{4\pi }ŝI\left(\overset{⇀}{r},t,ŝ\right)d^{2}ŝ.$$ (6)

Substituting Eq. (4) into Eq. (3) and integrating over all solid angles (using the assumption of isotropic radiance) yields a scalar term,

$$\frac{1}{v}\frac{\partial \Phi \left(\overset{⇀}{r},t\right)}{\partial t}+\nabla \cdot J\left(\overset{⇀}{r},t\right)+\mu_{a}\Phi \left(\overset{⇀}{r},t\right)=Q\left(\overset{⇀}{r},t\right),$$ (7)

and a vector term,

$$\begin{array}{ccc} \left(\frac{1}{v}\frac{\partial }{\partial t}+\mu_{a}+\mu_{s}-g\mu_{s}\right)J\left(\overset{⇀}{r},t\right)=-\frac{1}{3}\nabla \Phi \left(\overset{⇀}{r},t\right)+\int_{4\pi }q\left(\overset{⇀}{r},t,ŝ\right)ŝd^{2}ŝ, & & \end{array}$$ (8)

where $Q\left(\overset{⇀}{r},t\right)$ is the total power per volume radiating radially isotropically outward from the volume element at position $\overset{⇀}{r}$ and time *t* (in units of $\frac{\text{W}}{\text{c}{\text{m}}^{3}}$), and *g* is the ensemble average of the cosine of the scattering angle associated with a typical scattering event in the tissue [Fig. 5(a)]

$$g=\int_{4\pi }f\left(ŝ,{ŝ}^{\prime }\right)ŝ\cdot {ŝ}^{\prime }d^{2}{ŝ}^{\prime }=\left\langle \text{cos }\theta \right\rangle ,$$ (9)

where $f\left(ŝ,{ŝ}^{\prime }\right)$ is the scattering phase function which is the (wavelength-dependent) angular distribution of photons scattered from direction ${ŝ}^{\prime }$ to direction ŝ, *θ* is the angle between the incident and outgoing scattering wave vectors. This anisotropy factor *g* reflects the probability that a photon is scattered in the forward direction and in soft mammalian tissue typically has a value around 0.9. Though a full discussion of measurement and derivation of human tissue baseline optical properties is beyond the scope of this review, this is a fascinating topic of ongoing study, especially with regard to changes during early development (prenatal and postnatal) and atrophy with aging and disease.^47,74–88^ The reduced scattering coefficient can now be defined as $\mu_{s}^{\prime }=\left(1-g\right)\cdot \mu_{s}$ and, as described above, when combined with the absorption coefficient, is equal to the inverse of the transport mean free path (the random walk step). We can further simplify by assuming any sources are effectively isotropic

$$\int_{4\pi }q\left(\overset{⇀}{r},t,ŝ\right)ŝd^{2}ŝ=0.$$ (10)

If we now enforce a second key assumption (2) that variations in the photon current are slow relative to the time it takes the photons to travel a random walk step

$$\left|\frac{\partial J}{\partial t}\right|\ll v\left(\mu_{a}+\mu_{s}^{\prime }\right)\left|J\right|,$$ (11)

then Eq. (8) simplifies to a form similar to Fick’s law of diffusion

$$J\left(\overset{⇀}{r},t\right)=-\frac{1}{3\left(\mu_{a}+\mu_{s}^{\prime }\right)}\nabla \Phi \left(\overset{⇀}{r},t\right).$$ (12)

The constant of proportionality in Eq. (12), equal to one third of the transport mean free path, has units of length, whereas in Fick’s first law of diffusion, the constant of proportionality—the diffusion coefficient—has units of area per time. To maintain conceptual simplicity, we can define the photon diffusion coefficient (in units of $\frac{\text{c}{\text{m}}^{2}}{\text{s}}$) as

$$D\left(\overset{⇀}{r}\right)=\frac{v\left(\overset{⇀}{r}\right)}{3\left(\mu_{a}\left(\overset{⇀}{r}\right)+\mu_{s}^{\prime }\left(\overset{⇀}{r}\right)\right)},$$ (13)

where we are now explicitly noting that the index of refraction and the coefficient of absorption and reduced coefficient of scattering may vary in the tissue. Using this definition, we then substitute Eq. (12) into Eq. (7) to arrive at the diffusion approximation of the radiative transport equation for the photon fluence rate

$$\begin{array}{ccc} \frac{\partial \Phi \left(\overset{⇀}{r},t\right)}{\partial t}-\nabla \cdot \left(D\left(\overset{⇀}{r}\right)\nabla \Phi \left(\overset{⇀}{r},t\right)\right)+v\mu_{a}\left(\overset{⇀}{r}\right)\Phi \left(\overset{⇀}{r},t\right)=vQ\left(\overset{⇀}{r},t\right). & & \end{array}$$ (14)

In some cases, we can further simplify by assuming that the optical properties within the medium are spatially homogeneous. The diffusion equation in (14) then becomes

$$\frac{\partial \Phi \left(\overset{⇀}{r},t\right)}{\partial t}-D\nabla^{2}\Phi \left(\overset{⇀}{r},t\right)+v\mu_{a}\Phi \left(\overset{⇀}{r},t\right)=vQ\left(\overset{⇀}{r},t\right).$$ (15)

Equation (15) states that the temporal changes in the fluence rate are related to divergence due to diffusion (i.e., scattering), gains due to sources, and losses due to absorption. In practice, we often model the head using multiple tissue types (i.e., scalp/soft tissue, bone, gray matter, white matter, and cerebral spinal fluid), each with some set of estimated baseline optical properties.

To recap, the validity of the diffusion approximation for photon propagation in biological tissue is appropriate as long as (1) the radiance can be considered isotropic, which will generally be true in regions deeper than a mean free path, $l=\frac{1}{\mu_{a}+\mu_{s}^{\prime }}\approx 1.4\text{ mm}$; (2) the time scale of variations in fluence are much greater than the time it takes a photon to travel a mean free path $t_{l}=\frac{1}{v\left(\mu_{a}+\mu_{s}^{\prime }\right)}\approx 7\text{ ps}$ [Eq. (11)]; (3) the tissue properties are in the strong scattering regime ($\mu_{s}^{\prime }\gg \mu_{a}$, or, more concretely, $\mu_{s}^{\prime }>10\mu_{a}$); and (4) the source term $Q\left(\overset{⇀}{r},t\right)$ is isotropic.^89^ In the application of focus here, i.e., optical imaging of human brain function, these assumptions generally hold true at the depths of brain tissue. Though these assumptions break down in transparent or “void” regions of the head (e.g., within cerebral spinal fluid, CSF),^90,91^ the surface roughness of the boundaries between the CSF and surrounding layers enables these regions to be modeled using the diffusion approximation with effective optical properties to recover accurate reconstructions.^47,75,82,92^

The specific characteristics of the fluence rate response in Eq. (15) depend on the source term $Q\left(\overset{⇀}{r},t\right)$. Broadly, source terms utilized in human optical functional neuroimaging fall into three regimes: picosecond to nanosecond pulses (∼1 THz–1 GHz), intensity modulated light with frequencies in the ∼100 MHz–1 GHz range, and constant sources (essentially modulation below ∼1 MHz). The measurement types corresponding to these source modulation strategies are time domain (TD), frequency domain (FD), and continuous wave (CW), respectively. For the TD case, it can be shown^66,93^ that for a source term defined as a short isotropic pulse *Q*(*r*, *t*) = *δ*(0, 0) in an infinite and homogeneous medium, the solution to Eq. (15) becomes

$$\Phi \left(r,t\right)=\frac{vQ_{0}}{{\left(4\pi Dt\right)}^{3/2}}e^{\left[-\frac{r^{2}}{4Dt}-v\mu_{a}t\right]}.$$ (16)

This equation states that the distribution of the fluence rate around a point source is a spherically decaying Gaussian in space and exponential in time at large *r* [Figs. 5(b) and 5(c)]. The absorption relaxation time constant of this equation, $\tau =\frac{1}{v\mu_{a}}\approx 0.24\text{ ns}$ (corresponding to ∼4 GHz) for *λ* = 850 nm in gray matter tissue highlights the very short time scales required to adequately sample this fall-off distribution [Fig. 5(c)], also called the distribution of times of flight (DTOF) or the temporal point spread function (TPSF). TD systems use picosecond wide pulses of light sources and ultrafast optoelectronics to measure this temporal broadening of the detected light at some distance or set of distances from the source. The measured DTOF can then be fit to the expected distribution [Fig. 5(c)] to estimate underlying absorption and scattering properties of the tissue. As TD methods have yet to be fully realized in an HD-DOT array, a full discussion of TD methods and the exciting and rapidly advancing optoelectronics that enable these measurements^94–109^ are beyond the scope of this review.

For the case of intensity modulated light, the source term is written in the general form,^110–112^

$$Q\left(\overset{⇀}{r},t\right)=Q_{DC}\left(\overset{⇀}{r}\right)+Q_{AC}\left(\overset{⇀}{r}\right)e^{-i\omega t},$$ (17)

with both a DC and an AC component [Fig. 5(d)] where *ω* = 2*πf* is the angular frequency of the intensity modulation. In this case, Eq. (15) can be written in a simpler form by taking the Fourier transform of each term to get the frequency domain photon diffusion equation into the general form of the inhomogeneous Helmholtz equation

$$\left(\nabla^{2}+\kappa^{2}\right)\Phi \left(\overset{⇀}{r},\omega \right)=-\frac{v}{D}Q\left(\overset{⇀}{r},\omega \right),$$ (18)

where $\kappa^{2}=\frac{-v\mu_{a}+i\omega }{D}$. The solution to this equation in an infinite homogeneous medium with a modulated point source at the origin is given by the following overdamped solution to the wave equation,

$$\Phi \left(r,\omega \right)=\frac{vQ\left(\omega \right)}{4\pi D}\frac{e^{-k_{\text{Re}}r}e^{i\left(\omega t-k_{\text{Im}}r\right)}}{r},$$ (19)

where *r* is the distance from the modulated source and the wave vectors *k*_Re_ and *k*_Im_ are defined such that^72,110–112^

$$k_{\text{Re}}={\left[\frac{v\mu_{a}}{2D}\right]}^{1/2}{\left[{\left(1+{\left[\frac{\omega }{v\mu_{a}}\right]}^{2}\right)}^{1/2}+1\right]}^{1/2},$$ (20)

$$k_{\text{Im}}={\left[\frac{v\mu_{a}}{2D}\right]}^{1/2}{\left[{\left(1+{\left[\frac{\omega }{v\mu_{a}}\right]}^{2}\right)}^{1/2}-1\right]}^{1/2}.$$ (21)

Writing the wave vectors in this way highlights the length scale determined by the leading term of both wave vectors, ${\left[\frac{v\mu_{a}}{2D}\right]}^{1\;/2}\approx 1.4\text{ c}{\text{m}}^{-1}$ (assuming optical properties for gray matter and 850 nm photons as above). For modulation frequencies of *f* = 100 MHz and 1 GHz, the spatial attenuation lengths are $\frac{1}{k_{\text{Re}}}\approx 0.5\text{ cm and }0.4\text{ cm}$, and the diffuse photon density wave (DPDW)^63,113,114^ wavelengths are $\frac{2\pi }{k_{\text{Im}}}\approx 42\text{ cm and }4.9\text{ cm}$ [Fig. 5(d)]. For lower modulation frequencies where *ω* ≪ *vμ*_*a*_ → *f* ≪ 680 MHz (effectively, well below 100 MHz), the DPDW wavelength is so long that the phase difference Δ*θ*(*r*, *ω*) = *k*_Im_*r* measured at a detector some distance away from the source is too small to reliably measure. At very high modulation frequencies where *ω* ≫ *vμ*_*a*_, the phase becomes insensitive to the underlying optical properties; in fact, Eq. (11) can now be rewritten as $\omega \ll \frac{v^{2}}{3D}\to f\ll 25\text{ GHz}$ to provide an upper limit for modulation frequencies applicable to the diffusion approximation of the RTE.

Comparing the responses to a pulse [Eq. (16)] with the Fourier equivalent in the frequency domain [Eqs. (19)–(21)], we see that while the fluence rate of intensity modulated light propagates with a constant phase velocity $V_{ph}=\frac{\omega }{k_{\text{Im}}}$, the response to a pulse undergoes dispersion (pulse broadening in the time domain) due to the different phase velocity of each frequency component in the pulse [Figs. 5(c) and 5(d)]. Due to the significant cost of optoelectronics that maintain high fidelity in source modulation and photon detection at the required bandwidths for precise measurement of both light intensity and phase delay for FD methods, this strategy has yet to be implemented in HD-DOT arrays that require a high channel count of source-detector channels.

The CW regime can be modeled as the FD case with a modulation frequency of zero. This simplifies Eq. (18) to give the steady state diffusion equation

$$\left(\nabla^{2}-\frac{v\mu_{a}}{D}\right)\Phi \left(\overset{⇀}{r}\right)=-\frac{v}{D}Q\left(\overset{⇀}{r}\right),$$ (22)

which leads to the solution for the fluence rate in an infinite and homogeneous media

$$\Phi \left(r\right)=\frac{vQ_{0}}{4\pi D}\frac{e^{-r{\left[\frac{v\mu_{a}}{D}\right]}^{1/2}}}{r}.$$ (23)

In CW mode, only the magnitude of the light intensity is measured at the detector. In this case, because only one parameter is measured, relative changes in absorption in the modeled optical properties are all that can be accessed. By contrast, TD and FD systems, which measure the DTOF and both light intensity and phase relative to the source signal, respectively, provide access to relative (and, potentially, absolute) measures of absorption as well as scattering within the tissue. However, the current technology that supports these measurements is significantly more expensive and complex and has yet to be fully realized in an HD-DOT configuration.^101,115–124^ Therefore, Secs. II C–II E will focus primarily on the CW case.

### C. Perturbation methods: The Born and Rytov approximations

The solutions [Eqs. (16), (19), and (23)] to the diffusion approximation of the RTE [Eqs. (15), (18), and (22)] describe how photons propagate through turbid media given a constant background of steady-state optical properties. To address the goal of measuring changes in brain function within the volume (as manifested through changes in optical properties ***x***) via changes in the light signals ***y*** measured at the surface [as formalized as ***y*** = ***Ax*** in Eq. (1)], we must see how these solutions are altered given a small perturbation in optical properties. These perturbations are modeled as spatially varying deviations from their baseline values: $\mu_{a}\left(\overset{⇀}{r}\right)=\mu_{a}^{0}\left(\overset{⇀}{r}\right)+\delta \mu_{a}\left(\overset{⇀}{r}\right)$, and $\text{D}\left(\overset{⇀}{r}\right)=D^{0}\left(\overset{⇀}{r}\right)+\delta D\left(\overset{⇀}{r}\right)$, which are assumed to be small: $\delta \mu_{a}\left(\overset{⇀}{r}\right)\ll \mu_{a}\left(\overset{⇀}{r}\right)$ and $\delta D\left(\overset{⇀}{r}\right)\ll D\left(\overset{⇀}{r}\right)$, respectively. The resulting perturbations in fluence rate can be modeled using a simple linear expansion, also called the Born approximation, $\Phi \left(\overset{⇀}{r},{\overset{⇀}{r}}_{s}\right)=\Phi_{0}\left(\overset{⇀}{r},{\overset{⇀}{r}}_{s}\right)+\delta \Phi \left(\overset{⇀}{r},{\overset{⇀}{r}}_{s}\right)$, where $\delta \Phi \left(\overset{⇀}{r},{\overset{⇀}{r}}_{s}\right)\ll \Phi \left(\overset{⇀}{r},{\overset{⇀}{r}}_{s}\right)$.^73,118^ However, it has been shown that an exponential expansion in the fluence rate performs far better in practice and presents a much less ill-posed inverse problem, especially in cases of imaging deep perturbations (greater than ∼5 mm from the boundary)^73,118,125^

$$\begin{array}{ccc} \Phi \left(\overset{⇀}{r},{\overset{⇀}{r}}_{s}\right) & = & e^{\left[\Phi^{0}\left(\overset{⇀}{r},{\overset{⇀}{r}}_{s}\right)+\delta \Phi \left(\overset{⇀}{r},{\overset{⇀}{r}}_{s}\right)\right]}=e^{\Phi^{0}\left(\overset{⇀}{r},{\overset{⇀}{r}}_{s}\right)}e^{\delta \Phi \left(\overset{⇀}{r},{\overset{⇀}{r}}_{s}\right)}\\ & = & \Phi_{0}\left(\overset{⇀}{r},{\overset{⇀}{r}}_{s}\right)e^{\delta \Phi \left(\overset{⇀}{r},{\overset{⇀}{r}}_{s}\right)}, \end{array}$$ (24)

where the ${\overset{⇀}{r}}_{s}$ term denotes that these scattered fields are due to a spatially localized source, and $\delta \Phi \left(\overset{⇀}{r},{\overset{⇀}{r}}_{s}\right)\ll \Phi_{0}\left(\overset{⇀}{r},{\overset{⇀}{r}}_{s}\right)$, which then leads to a simple relationship for the perturbed fluence rate

$$\delta \Phi =\text{ln}\left(\frac{\Phi }{\Phi_{0}}\right).$$ (25)

This exponential expansion is referred to as the Rytov approximation. Experimentally, the Rytov approximation provides a means of normalizing such that small errors in assumed background optical properties divide out, thereby providing a more robust approach to imaging than the Born approximation.^73,118^ For the general case of a complex fluence rate, *Φ* = *Ae*^*iθ*^, the Rytov approximation provides a relationship for the perturbed fluence rate that automatically separates attenuation in light amplitude from phase shifts between the incident $\Phi_{0}=A_{0}e^{{i\theta }_{0}}$ and measured *Φ* = *Ae*^*iθ*^ signals

$$\ln\left(\frac{\Phi }{\Phi_{0}}\right)=\ln\left(\frac{Ae^{i\theta }}{A_{0}e^{{i\theta }_{0}}}\right)=\ln\left(\frac{A}{A_{0}}\right)+i\left(\theta {-\theta }_{0}\right).$$ (26)

Again, we see that the CW case is simply a special case of the FD case (i.e., there is no phase term to consider). The baseline fluence rate $\Phi_{0}\left(\overset{⇀}{r},{\overset{⇀}{r}}_{s}\right)$ is assumed to arise from some baseline spatial distribution of optical properties $\mu_{a}^{0}\left(\overset{⇀}{r}\right)$ and $D^{0}\left(\overset{⇀}{r}\right)$ within the volume [Fig. 3(b)]. When imaging functional brain activity, the baseline fluence rate for a given source-detector measurement pair is typically estimated using the temporal mean of the time course of that measurement during an experiment [i.e., $\Phi_{0}\left({\overset{⇀}{r}}_{d},{\overset{⇀}{r}}_{s}\right)=\Phi_{0,sd}=\left\langle \Phi_{sd}\left(t\right)\right\rangle$]. Alternatively, the baseline can be estimated from a time period immediately preceding an experimental induction of a perturbation via some task (e.g., a Valsalva maneuver).

### D. Numerical methods

The above solutions to the diffusion approximation of the RTE are all analytically derived given the infinite model and homogeneous optical properties. For calculating solutions to the diffusion equation for tissues with an arbitrary and complex geometry (i.e., a head) and spatially varying optical properties (index of refraction, absorption, and scattering coefficients), a number of publicly available packages such as NIRFAST^126^ or TOAST++^127^ are available. These packages utilize powerful, flexible, and fast finite element modeling (FEM) routines.^128–130^ Alternatively, Monte Carlo methods can be employed.^131–134^ A strength of Monte Carlo methods is that they do not rely on assumptions of isotropic radiance or slow changes in photon currents. Additionally, Monte Carlo methods provide solutions with greater accuracy within the top millimeter surface of the tissue—where the assumptions required for the diffusion approximation break down and can lead to numerical errors. However, Monte Carlo methods are comparatively slow relative to FEM methods that rely on the diffusion approximation. At the depth of the human brain the diffusion approximation works quite well and is computationally far more efficient, allowing for solutions to be obtained for thousands of source-detector measurement pairs in a complex head geometry in just a few minutes.^55,135^

The solutions to these problems can be derived using the methods of Green’s functions where $G\left({\overset{⇀}{r}}_{s},\overset{⇀}{r}\right)$ and $G\left(\overset{⇀}{r},{\overset{⇀}{r}}_{d}\right)$ are the Green’s functions for the sources and detectors located at positions ${\overset{⇀}{r}}_{s}$ and ${\overset{⇀}{r}}_{d}$, respectively [Figs. 3(c) and 5(e)]. The Green’s function represents the spatial sensitivity of a given source or detector. Importantly, though the exact functional form of a Green’s function depends upon the geometry of the problem, the Green’s function for a source or a detector are of the same functional form, as derivable from the reciprocity theorem of electromagnetic radiation.^136^ This fact directly gives rise to the adjoint formulation of the sensitivity relations.^73^ Reciprocity essentially states that transmitters and receivers of electromagnetic radiation can be equivalently modeled. Thus, $G\left({\overset{⇀}{r}}_{s},\overset{⇀}{r}\right)$ and $G\left(\overset{⇀}{r},{\overset{⇀}{r}}_{d}\right)$ provide a measure of some “effect” at voxel $\overset{⇀}{r}$ in the tissue due to a source at position ${\overset{⇀}{r}}_{s}$ or an “adjoint source” at position ${\overset{⇀}{r}}_{d}$ [Fig. 5(e)].

Solving the simpler CW case [Eq. (22)] using the Rytov approximation with Green’s function methods, neglecting terms beyond the first order, and relating to changes in detected intensity at the surface ***y*** (also referred to changes in optical density) leads to the following solution:

$$\begin{array}{ccc} y\left(\lambda \right)=-\ln\left(\frac{\Phi }{\Phi_{0}}\right)=-\frac{v}{D}\int \frac{G\left({\overset{⇀}{r}}_{s},\overset{⇀}{r},\lambda \right)\cdot G\left(\overset{⇀}{r},{\overset{⇀}{r}}_{d},\lambda \right)}{G\left({\overset{⇀}{r}}_{s},{\overset{⇀}{r}}_{d},\lambda \right)}\Delta \mu_{a}\left(r\right)d\overset{⇀}{r}. & & \end{array}$$ (27)

This equation states that ratiometric (i.e., differential) measurements of fluence at the boundary are related to the spatial distribution of internal changes in absorption multiplied by the spatial wavelength-dependent sensitivity distributions for the source and detector and summed over all points in the tissue. The normalization term within the integral (sometimes referred to as *G*_*sd*_) is the Green’s function of the source evaluated at the position of the detector. Here, ***y*** is a vector with each element corresponding to a specific source-detector pair at a given wavelength.

The next step is to discretize Eq. (27) for some finite set of *N*_*m*_ source-detector pair measurements over a set of *Nv* voxels or nodes within a finite element mesh [Fig. 3(b)]. Using small-volume voxels (tetrahedral elements for the mesh) will facilitate more accurate solutions but will also add to the computational time required.^135^ While it is true that DOT is a relatively low-resolution imaging modality, it is important that the forward model be accurate enough that image quality of the data is not compromised due to discretization errors in the model.^46,55,135^ To achieve fMRI-comparable image quality, it is recommended that the tetrahedral elements have a volume of ∼1–1.5 mm^3^ each (which typically requires 800 000–1 000 000 nodes total in a head mesh).^46,47^ Equation (27) can be rewritten as

$$y\left(\lambda \right)=-\frac{vV}{D}\sum_{j}^{N_{v}}\frac{G\left({\overset{⇀}{r}}_{s},{\overset{⇀}{r}}_{j},\lambda \right)\cdot G\left({\overset{⇀}{r}}_{j},{\overset{⇀}{r}}_{d},\lambda \right)}{G\left({\overset{⇀}{r}}_{s},{\overset{⇀}{r}}_{d},\lambda \right)}\Delta \mu_{a}\left(r_{j}\right),$$ (28)

where *V* is the volume of the discretization element. This equation can be rewritten in a format where $y\left(t\right)=-\ln\left(\frac{\Phi \left(t\right)}{\Phi_{0}}\right)$ is the vector of optical density changes for each of the source-detector measurement pairs (each as a function of time), ***A*** is the sensitivity matrix derived from the full light model, and ***x*** = Δ*μ*_*a*_(***r***_***j***_, *t*) is a vector representing the change in absorption in each voxel (also a function of time)

$$y\left(t\right)=Ax\left(t\right).$$ (29)

For simplicity, it is assumed here that the sensitivity is itself not a function of time. In practice, the measurements, the absorbance, and the sensitivity matrix are each a function of the wavelength of light emanating from the sources.

In the FD case, as shown in Eq. (26), the Rytov approximation naturally separates the amplitude and phase components of the measurements (written here as vectors to account for multiple sources, detectors, wavelengths, and modulation frequencies) into real and imaginary components of $y\left(\lambda ,\omega \right)=-\ln\left(\frac{\Phi }{\Phi_{0}}\right)=\left[\begin{array}{c} Y_{Re}\\ Y_{Im} \end{array}\right]=\left[\begin{array}{c} Y_{A}\left(\lambda ,\omega \right)\\ Y_{\theta }\left(\lambda ,\omega \right) \end{array}\right]$, where $Y_{A}\left(\lambda ,\omega \right)=\ln\left(\frac{A}{A_{0}}\right)$ and ***Y***_***θ***_(**λ**, **ω**) = *θ* − *θ*_0_. Similarly, the perturbations in optical properties are separable $x=\left[\begin{array}{c} \Delta \mu_{a}\left(r\right)\\ \Delta D\left(r\right) \end{array}\right]$. The full FD sensitivity matrix contains four separable components corresponding to the real and imaginary components of sensitivity of measurements at the surface to internal changes within the volume of absorption and scattering $A=\left[\begin{array}{cc} Re\left(W_{\Delta \mu_{a}}\right) & Re\left(W_{\Delta D}\right)\\ Im\left(W_{\Delta \mu_{a}}\right) & Im\left(W_{\Delta D}\right) \end{array}\right]$, where the complex sensitivity relations for a given source-detector pair measurement in relation to absorption Δ*μ*_*a*_ and scattering Δ*D* are given by^125,137,138^

$$W_{\Delta \mu_{a}}\left(\lambda ,\omega \right)=-\frac{vV}{D}\frac{G\left({\overset{⇀}{r}}_{s},\overset{⇀}{r},\lambda ,\omega \right)\cdot G\left(\overset{⇀}{r},{\overset{⇀}{r}}_{d},\lambda ,\omega \right)}{G\left({\overset{⇀}{r}}_{s},{\overset{⇀}{r}}_{d},\lambda ,\omega \right)},$$ (30)

$$W_{\Delta D}\left(\lambda ,\omega \right)=\frac{vV}{D}\frac{\nabla G\left({\overset{⇀}{r}}_{s},\overset{⇀}{r},\lambda ,\omega \right)\cdot \nabla G\left(\overset{⇀}{r},{\overset{⇀}{r}}_{d},\lambda ,\omega \right)}{G\left({\overset{⇀}{r}}_{s},{\overset{⇀}{r}}_{d},\lambda ,\omega \right)}.$$ (31)

The FD measurements and Green’s functions all depend on both the wavelength λ and modulation frequency ω of the incident light.

To calculate the light model ***A***, many labs use NIRFAST^126^ to model the Green’s functions [Figs. 3(c), 5(d), and 5(e)], which are primarily dependent upon three things: (1) the tissue boundary shape, (2) the internal distribution of baseline optical properties, and (3) the locations of the sources and detectors on the surface [Fig. 3(b)], as well as the wavelength and (in the FD case) the modulation frequency. The tissue shape and optical property distributions are ideally generated from a subject-specific segmentation of the head,^47,139,140^ though atlas-based models can work quite well when subject-specific anatomy is not available.^44,52,87^

### E. Image reconstruction

As described above, the sensitivity matrix relates relative ratiometric changes in light-level measurements taken at the surface to relative changes in absorption within the volume. The sensitivity matrix can be directly inverted for image reconstruction using Tikhonov regularization along with spatially variant regularization to minimize the objective function [Fig. 3(e)]

$$\text{min}\left\{{∥y_{meas}-Ax∥}_{2}^{2}+\lambda_{1}{∥Lx∥}_{2}^{2}\right\}.$$ (32)

The penalty term for image variance, $\lambda_{1}{∥Lx∥}_{2}^{2}$, incorporates a spatially variant regularization term *λ*_2_ where^45,143^

$$\text{diag}\left(L\right)=\sqrt[{2}]{\left[\text{diag}\left(A^{T}A\right)+\lambda_{2}\right]}.$$ (33)

The specific values of these parameters will directly influence the DOT imaging domain characteristics [as visualized in Fig. 3(c–iv)] and should be considered aspects of the system design along with the hardware. A solution,

$$x=A_{\lambda_{1}\lambda_{2}}^{\#}y_{meas},$$ (34)

can thus be directly obtained using a Moore-Penrose generalized inverse with

$$A_{\lambda_{1}\lambda_{2}}^{\#}=L^{-1}{\left({Ã}^{T}Ã+\lambda_{1}I\right)}^{-1}{Ã}^{T}y_{meas},$$ (35)

where,

$$Ã=\tilde{A}L^{-1}.$$ (36)

The optimal values of regularization parameters *λ*_1_ and *λ*_2_ depend upon the source-detector grid geometry, the underlying noise characteristics of the imaging system, and the geometry of the anatomical model. The Tikhonov regularization term *λ*_1_ tunes the balance between amplifying high spatial frequency information (including noise) at small values (typically below 0.01, though the exact number depends on the number of measurements in the imaging system) and strongly weighting low-spatial-frequency modes which effectively spatially smooth the image domain at large values.^141^ The spatially variant regularization term *λ*_2_ has been shown to improve localization error and to provide a more uniform resolution and contrast within the imaging domain and thereby an improvement in image quality of DOT reconstructions.^117,142–145^ As the sensitivity of HD-DOT drops off with depth from the surface [Figs. 3(c), 5(b)–5(e)], spatially variant regularization provides a way to tune the reconstruction to an appropriate depth; too small of a *λ*_2_ will push the reconstruction too deep below the surface and too large a value will pull the reconstruction too shallow. Optimal settings for these parameters are found through simulation and empirical studies to provide uniform imaging across the field of view as judged by evaluating point spread functions (in simulation) and, ideally, subject-matched comparisons to an alternate modality, such as functional MRI. An estimate of the spatial extent of the imaging domain can be found by calculating and visualizing a flat field reconstruction. This done by generating a test image *∂****x*** of a global unit change in absorption throughout the imaging volume (the ‘flat field’ perturbation) to generate simulated data ***y***_*sim*_ via

$$y_{sim}=A\partial x.$$ (37)

The flat field reconstruction of the imaging domain ***x***_***ff***_ is then found as in Eq. (34)

$$x_{ff}=A_{\lambda_{1}\lambda_{2}}^{\#}y_{sim}.$$ (38)

The spatial profile of this flat field reconstruction provides a visual readout of the smoothness and extent of the imaging domain throughout the volume. Where the flat field lies below 1%–10% informs where should not be considered valid in volumetric reconstructions.^33,46,55,126,146^

Relative changes in hemoglobin concentrations ******Δ***C*** can then be obtained from the absorption coefficients used in spectral decomposition

$$\Delta C=E^{-1}\Delta \mu_{a},$$ (39)

where ***E*** is a matrix containing the extinction coefficients of HbR and HbO_2_, and ******Δ***C*** = [*Δ*[*HbO*_2_], *Δ*[*HbR*]] is the matrix of concentration changes by time.

## III. HIGH-DENSITY DIFFUSE OPTICAL TOMOGRAPHY SYSTEM DESIGN

**FIG. 6.**
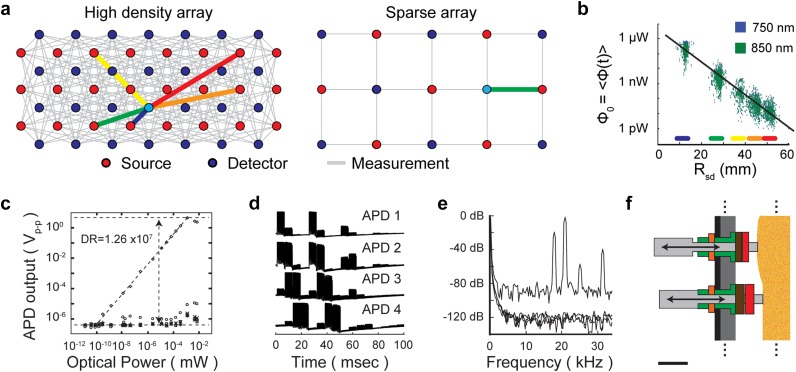
A high density grid design presents multiple electronic and mechanical challenges. (a) The high density grid provides overlapping measurements at multiple source-detector distances, thereby supporting many times more optical measurements per surface area of the head than a sparse array. Colored thick lines denote associated source-detector distances as marked in part b. The high density array shown here supports source-detector distances of 1.3 (blue), 3.0 (green), 3.9 (yellow), 4.7 (orange), and 5.1 (red) cm. (b) To accurately detect light from sources at multiple distances, the detectors must have a linear response over many orders of magnitude, a low noise floor, and low crosstalk. (c) Discrete avalanche photo diode (APD) detection channels provide adequate dynamic range for the demands of the HD-DOT system. (d) Time encoding strategies minimize crosstalk between detection channels. (e) Frequency and region encoding strategies provide additional tools for minimizing crosstalk while allowing for time-efficient sampling of physiological dynamics. (f) The density of source and detector fibers (or optical elements) at the scalp lead to significant challenges in maintaining optical coupling. Successful imaging cap designs often use semi-rigid thermal plastic to maintain a regular grid along with flexible spring-like action on the fibers to facilitate combining through the hair. [Adapted with permission from Eggebrecht *et al.*, Nat. Photonics **8**, 448–454 (2014). Copyright 2014 Springer Nature Publishing.]

Accurate reconstruction of relative changes in hemodynamics fundamentally depends upon obtaining high fidelity signal quality of light levels from multiple overlapping measurements that are separated by multiple distances (Fig. 6). This key requirement directly leads to challenges in the optoelectronics and challenges in maintaining good optical coupling throughout the system—from the source to the scalp and from the scalp to the detector. The large number of independent source-detector measurements also presents significant challenges in real-time data quality assurance. Each of these sets of challenges will be discussed below.

### A. Challenges in optoelectric designs

Source-detector measurement pairs at multiple separations provide additional depth information, and the overlapping measurements at a given separation support improved lateral resolution.^45,147–149^ For example, the array shown in Fig. 6(a) [equivalent to a subset of that in Fig. 3(a)] utilizes measurements separated by distances of 1.3, 3.0, 3.9, 4.7, and 5.1 cm for the first five nearest neighbor separations, which leads directly to significant challenges in obtaining an adequate dynamic range of response for each detector while minimizing crosstalk between detection channels. Maximizing the dynamic range while minimizing crosstalk involves multiple system design considerations: the light budget, the detection and amplification strategy, and encoding/decoding strategies.

#### 1. The light budget

The source type, be they light emitting diodes (LEDs) or laser diodes (LDs), will significantly impact system design. First, the choice of wavelengths for the sources may be motivated by spectral width considerations, as LEDs emit photons over a relatively broad band around their characteristic center wavelength relative to LDs. Additionally, though LDs can be modulated faster than LEDs, LDs are typically not available at as many wavelengths as LEDs. The optimal choice of wavelengths, and optical bandwidths of sources will depend on the required spectroscopy for the specific goals of the application,^24,150–152^ be it imaging hemoglobin, cytochrome c oxidase,^153–156^ or other functional chromophores. Each source position of the system highlighted in Fig. 3 uses LEDs emitting 750 nm and 850 nm photons with an optical power at the head of 3.2 ± 0.3 mW and 4.3 ± 0.3 mW for each LED.^46^ Other systems have used different wavelength combinations including 760 and 830 nm (the DYNOT 232 optical tomography imager of NIRx^59^), 690 and 830 nm (the ISS Imagent™^157^ and the CW4 TechEn, Inc. system^44^), 660, 780 and 850,^158^ and even a larger set of wavelengths including 778, 808, 814, 841, 847, 879, 888, and 898 nm.^159,160^ The system in Figure 3 used three 750 nm LEDs per channel to compensate for the strong attenuation in biological tissue at that wavelength. Though one can increase the intensity of the source, the American National Standards Institute (ANSI) limits the amount of NIR light deposited on human tissue at a maximum intensity of 4 mW/cm^2^ at these wavelengths. This specification of source intensity at the scalp sets an upper limit on the light signal intensity to be collected from the head some distance away.

The moment light leaves the source, losses occur due to poor coupling between the LED/LD and the fiber optic, loss along the fiber optic (if the fiber is made with a lossy material like plastic or if the fiber has been broken), and poor coupling at the scalp. Coupling between the source and a fiber optic depends on not just the optical alignment, but also the etendue of both the source and the fiber. The etendue of an optical element is equal to the area of emission (or collection) times the solid angle of emitted (or collected) light. When comparing coupling designs, and when the optics are axially symmetric, the solid angle can be roughly approximated by the square of the numerical aperture (NA) of the fiber optics. Also important with fiber optics is whether or not the fiber is a single-core fiber or a fiber bundle. Larger fibers provide easier optical coupling, however, they are stiffer than smaller fibers that can be challenging to align reliably. Fiber bundles that pack many small optical fibers into a single larger conduit provide a reasonable middle ground for many designs. Because the individual glass fibers are smaller, fiber bundles tend to be more forgiving to breaking (i.e., smaller fibers have a smaller critical bend radius). However, the price one pays for using a fiber bundle is found in the packing fraction: one can expect to lose a significant fraction of the light impingent on the fiber (typically up to 50%) because there are gaps in between the small glass fibers that will not transmit the light. Similar concerns are present on the return path of the photons into the detector. Exciting new advances in on-the-head optoelectronic components remove the fibers from the design, which simplifies some system design considerations.^159,160^ However, challenges remain in reliable maintenance of power consumption, data streaming fidelity, and participant comfort.

#### 2. Detection and amplification

Over the NIR wavelength range, light levels at source-detector distances from 1-5 cm vary over at least six orders of magnitude in optical power [Figs. 6(a) and 6(b)]. To ensure a linear output over such a range of optical power inputs, many HD-DOT systems use avalanche photodiodes (APD) that can be sourced from various distributors including, e.g., Hamamatsu.^25,45,46^ The APD design is generally preferred over a photon multiplier tube (PMT) design due to the strong demands on dynamic range, though some systems successfully implement PMTs.^157^ The APDs provide a dynamic range of up to >10^7^ [Fig. 6(c)], which allows for a signal to noise level (SNR) > 100 over 4–5 orders of magnitude in light level.^46^ This high level of SNR is crucial because changes in hemodynamic-measured brain function due to task activations is of order a few percent and variance in the resting state is of order 1% or less.^47,161,162^

In addition to dynamic range, additional key specifications when optimizing the detection strategy include the sensitivity, noise equivalent power (NEP), and crosstalk. The sensitivity, the ratio of output voltage for a given input optical power, should typically be at least 1 × 10^6^ V/W. The NEP, the optical input-referred power of the noise floor output of a detector, should be as small as possible (e.g., less than 20 fW/√Hz). Crosstalk is a measure of how much interference a signal in one source-detector channel has on a separate source-detector channel. Constraints on the levels of allowable crosstalk are driven by the requirements in dynamic range: to ensure data is uncorrupted over a dynamic range of 120 dB, the crosstalk must be kept below −120 dB. Electronic crosstalk between detection channels can occur through common power supplies or within a multichannel analog to digital converter (ADC). To maintain these specifications, systems typically use avalanche photodiodes coupled into 24-bit dedicated ADCs.^27,45,46^ Many companies provide commercially available high fidelity ADCs including MOTU, RME, and Focusrite. Additional strategies for increasing the effective dynamic range can be employed that use dynamic gain adjustments before the signal reaches the ADC. These strategies can be complex and can lead to poor crosstalk performance compared to the strategies described above.^57,163,164^

#### 3. Encoding/decoding

To maintain low crosstalk between source-detector measurement channels, also requires encoding and decoding strategies. Time encoding [Fig. 6(d)] along with frequency and spatial encoding [Fig. 6(e)] may be employed.^46^ In time encoding, only the source light at a given position is turned on at a given time, here called a time step. This minimizes potential crosstalk between different source-detector pair measurement channels because it is straight forward to assign the signal for every detector to the exact source that is on. However, this strategy can be slow and lead to under sampling physiology if there is a larger number of sources to encode. To obtain a faster frame rate (i.e., the time is takes to sample the entire field of view), frequency encoding may be employed whereby multiple sources are modulated at the same time, but at different frequencies [Fig. 6(e)]. Then the signal for a given source-detector pair is obtained via a Fourier decomposition of a given detector’s data within a time step where the magnitude of the signal from a source is proportional to the magnitude at its modulation frequency. With frequency encoding multiple sources can be on at once as long as the Fourier peaks are far enough apart that they do not overlap, otherwise crosstalk between those respective channels will go up significantly. Additionally, with multiple sources on, broad band shot noise will contaminate the Fourier spectrum [see raised noise floor in trace with peaks in Fig. 6(e)]. A higher level of overall light will effectively lower the dynamic range for the source-detector measurements. One can also spatially encode the sources such that spatially separated sources on the HD array are on at the same time. One must be careful that the shot noise from very bright sources is not swamping out the desired light from more distant sources in a given encoding strategy. The system highlighted in Fig. 3 uses a combination of time, frequency, and spatial encoding.^45,47^ With each of these encoding strategies, it is important to note that background light levels from the room or immediate imaging environment may lead to significant crosstalk and a loss of dynamic range. The Fourier decomposition strategy of decoding provides a robust strategy to minimize the effects of background light level. These strategies should be implemented with care for the desired frame rate: sampling too slowly can lead to aliasing of physiology variance into the data stream. A minimal frame rate of 3 Hz (optimal if 10 Hz or faster) is recommended to allow for adequate sampling of systemic physiology which includes both respiration (generally around 0.3 Hz) and pulse (generally around 1 Hz for a quietly resting healthy adult).

### B. Challenges in optode-scalp coupling and cap design

**FIG. 7.**
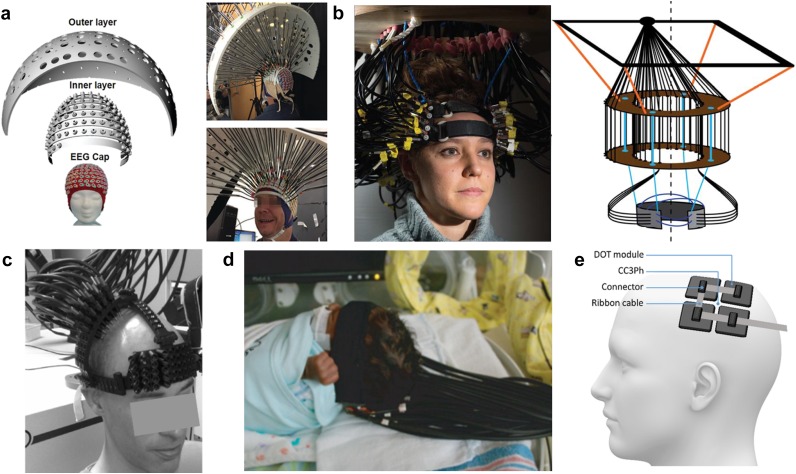
Strategies for HD-DOT cap design and fiber management. (a) An outer layer manages fiber weight and position and directs the fibers toward an inner layer via plastic tubing. [Reproduced with permission from Dai *et al.*, J. Biophotonics **11**, e201600267 (2017). Copyright 2017 John Wiley and Sons.] (b) A “double-halo” design supports the weight of the 188 optical fibers while allowing moderate planar motion of the participant. This design minimizes torque on the fibers at the scalp surface which effectively dampens motion artifact in the acquired signal. The imaging cap is attached to the head of the participant using adjustable hook-and-loop straps across the forehead and over the top of the head. [Reproduced with permission from Eggebrecht *et al.*, Nat. Photonics **8**, 448–454 (2014). Copyright 2014 Springer Nature Publishing.] (c) An open scaffolding design using semi-plastic strips placed along the head circumference along the midline and just above the ears. This design uses spring-loaded fibers to maintain optimal coupling at the fiber-scalp interface. [Reproduced with permission from Koch *et al.*, Front. Neuroenerg. **2**, 12 (2010). Copyright 2010 Frontiers.] (d) Bedside HD-DOT imaging across bilateral visual cortex in infants in the hospital neonatal ward. The soft silicone array surrounds each source and detector tip and provides a comfortable surface on which to rest the infant’s head. Straps composed of neoprene with hook-and-loop connections secure the silicone array to the head with the bed supporting the weight of the fibers. [Reproduced with permission from White *et al.*, NeuroImage **59**, 2529 (2012). Copyright 2012 Elsevier.] (e) A fiberless modular HD-DOT system transmits data via a ribbon cable to a controller, significantly increasing wearability of the technology. [Reproduced with permission from Chitnis *et al.*, Biomed. Opt. Express **7**, 4275–4288 (2016). Copyright 2016 OSA Publishing.]

Beyond challenges in optical and electrical components, reliable and consistent coupling of the optical elements to the scalp of the participant presents multiple significant challenges. Sources and detectors may be placed directly on the head^165^ or coupled via optical fibers.^45,166,230,231^ A general principle in ensuring reliable and comfortable imaging arrays is to provide a lightweight but rigid structure that maintains the optical fiber positions while minimizing torque on the fibers that can lead to coupling inconsistency over the course of an imaging session. For example, several adult DOT systems have used a rigid outer shell to manage fibers and bear fiber weight [Figs. 6(f) and 7]. Other DOT systems image the participants (mostly infants in the current literature) in the supine position so that the bed bears the weight of the fibers [Fig. 7(d)]. A combination of foam and elastic pieces can help maintain a force perpendicular to the head surface to hold the optodes directly coupled against the scalp while allowing for moderate translation normal to the head such that the imaging cap can conform to local variations in head shape [Fig. 6(f) used in cap design Fig. 7(b)].^26,46^ Alternatively, a spring loaded fiber tip can couple fibers to the scalp.^59^ Furthermore, rigid outer structures aid in fiber management and suspend the weight of the fibers.^158^ Recent work has designed more wearable caps with lightweight fibers.^167^ Finally, recent developments in wireless systems have minimized the need for fiber management and weight bearing designs [Fig. 7(e)].

**FIG. 8.**
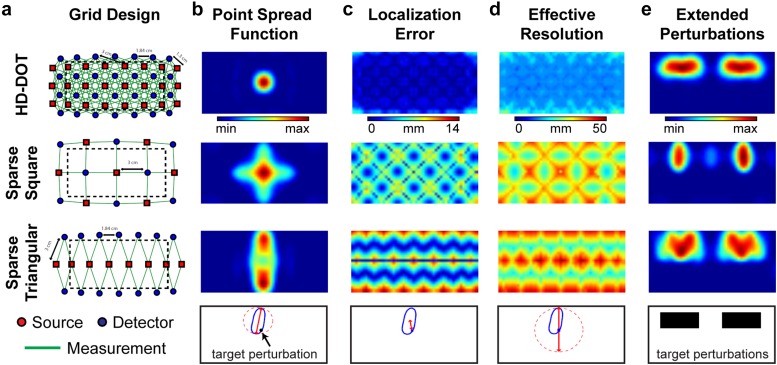
Modeling cap design and resultant simulated data quality. (a) A regular HD-DOT grid design with 24 sources and 28 detectors supported 212 source-detector measurements using both first and second nearest neighbor distances of 13 mm and 30 mm. Sparse grid designs covering the same surface area include a square design (7 sources, 8 detectors, 22 measurements) and a triangular design (8 sources, 14 detectors, 28 measurements) have access to only source-detector pairs separated by 30 mm. (b) A modeled point spread function for each grid design highlights the spatial irregularity of reconstructed data with the sparse grids as compared to the HD-DOT grid. (c) Localization error (the spatial separation between the known target location and the centroid of the reconstructed voxels) demonstrates that sparse square and triangular grids have higher localization error than HD-DOT. (d) The effective resolution was calculated as the diameter of a circle centered at each target position needed to enclose the reconstructed response. These metrics highlight both the superior resolution and the regularity of the reconstructions of the HD array across the imaging domain. (e) Reconstructions of extended perturbations further highlight irregularities of reconstructions using sparse grids as compared to an HD array. [Adapted with permission from B. R. White and J. P. Culver, J. Biomed. Opt. **15**, 026006 (2010). Copyright 2010 SPIE.]

A further consideration is the choice of source-detector layout. Sparse DOT grids (i.e., source detector separation distance >15 mm) will give rise to systematic data quality perturbations based upon the respective point spread function (Fig. 8). The size, shape, and severity of artifacts in the point spread function of the observed data will depend upon the cap design (e.g., square, triangular, rectangular, HD), and metrics of image quality such as localization error and effective resolution will spatially vary in a systematic fashion based upon source-detector distance and location. For example, Chance and colleagues note that their chosen source-detector layout resulted in elongated activations where fMRI demonstrated localized activity.^168^ Sparse square and triangular grids will have greater localization error and worse effective resolution than HD-DOT cap designs^149^ (Fig. 8).

A crucial design detail, regardless of whether fibers or fiberless designs are used, is to comb through the participant’s hair to gain unimpeded access to the scalp [e.g., as in Fig. 6(c)]. Hair (and product in hair like conditioner or hair gel) scatters light away from the optic-head system and lowers raw data quality. With this infrastructure in place, the cap maintaining the imaging array may be attached to the participant with hook-and-loop straps positioned to provide rigid yet comfortable stability on the head and to conform the curvature of the cap to a wide variety of head shapes and sizes. To ensure consistent placement of the imaging array on a given participant and across participants, measurements of the distance between specific fiducials on the imaging array and the head of the participant (e.g., the nasion, left and right tragus, and eyes) should be recorded.

### C. Challenges in data quality assurance

To provide adequate coupling across the imaging array, a few simple metrics of data fidelity can help ensure a high quality cap fit. First, the average light level for each source and detector can be displayed in a two-dimensional representation of the imaging array [Fig. 3(d–ii)]. If the light level is low, or if there is significant spatial variance in mean light level (more than 2 orders of magnitude), then the associated optical element or fiber optic should be adjusted at the head to improve coupling. The adjustment typically involves improving either the combining through the hair and/or ensuring the fiber/element is coupled to the scalp at a right angle. Second, adequately coupled elements will reflect a set of mean light levels that are logarithmically distributed as a function of distance, reflecting diffusion of photons through tissue [Fig. 6(b) and Eq. (23)]. Third, if the spread in light level at a given source-detector separation is more than 1–2 orders of magnitude, or if the slope of the fall-off is not approximately one order of magnitude in light level for every centimeter of additional Rsd, then the cap fit may not be optimal. Third, assuming the data are acquired at a frame rate of at least 3 Hz, the time course of individual source-detector pair measurements with a good signal-to-noise ratio will clearly exhibit characteristics consistent with the pulse (∼1 Hz) frequency [Fig. 3(d–iii)]. The relative magnitude of the pulse peak in a power spectral density plot is an excellent indicator of data quality: the more noise contamination, the lower the relative pulse peak power.

## IV. VALIDATION

As is true with any technology development, external validation of the acquired signals provides essential corroboration required for establishing that the technology delivers meaningful information that complements existing measurement strategies. Validation of optically-measured neurophysiological signals and the anatomical specificity of the reconstructed maps is a necessary and crucial step toward adoption of the method beyond the optical community. For HD-DOT, validation studies have used simulation and *in vivo* direct and indirect comparisons against fMRI as a gold-standard of functional neuroimaging. Well-understood task-based paradigms that elicit reliable responses in sensory and motor areas provide solid footing for cross-modal validation because the brain responses from these tasks are more predictable and less variable across a population than tasks designed to elicit responses in cognitive brain areas. Task-free paradigms that leverage the spatial structure of temporal correlations of very low frequency activity within the brain (i.e., functional connectivity) provide a more stringent bar for validation due to increased demands of the instantaneous signal-to-noise. Sections IV A–IV D will discuss some key validation-focused studies that have established HD-DOT as an effective and reliable neuroimaging tool producing cortical brain maps with comparable precision to fMRI in both adults and infant participants.

### A. Validation of HD-DOT with retinotopy paradigms

Retinotopy, so named because visual stimuli incident on the retina map onto visual cortex in a regular and characteristic pattern,^169^ provides a compelling strategy for optical imaging validation for multiple reasons. First, the spatial organization of retinotopic maps at multiple spatial scales is well known via investigation with PET,^170^ fMRI,^171–178^ and other methods.^179–183^ Second, retinotopic organization can be reliably measured within an individual.^171,174,184^ Third, the detailed spatial structure of the retinotopic maps vary between individuals, providing opportunity for demonstration of image quality at ever finer spatial scales.^185^ Fourth, because retinotopic maps are in a primary sensory region of the brain, interpretation of the measured responses is simpler than in regions of cortex that support higher cognitive functions. Indeed, mapping visual fields in occipital cortex was one of the earliest forms of fMRI methods validation against PET imaging.^171,172,177,186–191^ Following in this tradition, several DOT studies have utilized retinotopy to establish proof of principle, validity, and reliability of the technique for imaging human brain function in adults^45,47,48,192,193^ and even in infants.^166^

**FIG. 9.**
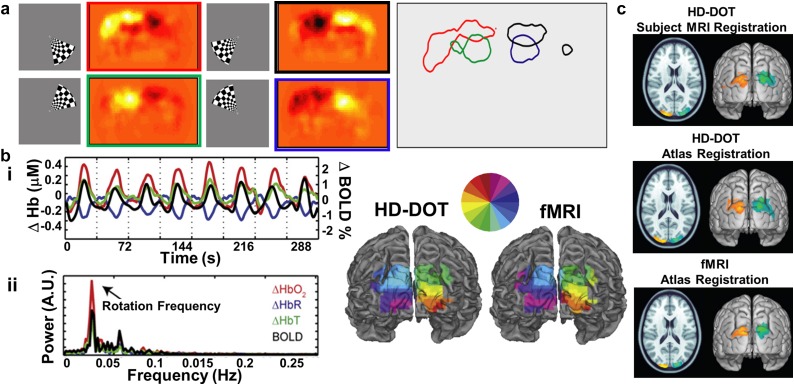
Validation of HD-DOT image quality using *in vivo* retinotopy. (a) Flickering checkerboard wedges and corresponding activations in four quadrants of the visual field recorded in a single subject. As expected, HD-DOT reconstructs brain activations in the opposite quadrant as the visual stimulation (e.g., red: bottom right visual field stimulation causes activation in top left visual cortex as viewed from behind the head). Contours on right denote 50% maximum of activations. [Adapted with permission from Zeff *et al.*, Proc. Natl. Acad. Sci. U. S. A. **104**, 12169–12174 (2007). Copyright 2007 National Academy of Sciences.] (b) Rotating the flickering wedge around the field of view extends the quadrant design to a phase encoded retinotopy design wherein the spatial location of the stimulus is encoded in the temporal phase of the response. (i) The periodic response to the phase encoded design in a single voxel in the visual cortex of a single subject. (ii) The power spectrum of that response exhibits a strong peak at the stimulation frequency. Mapping the phase at the stimulus frequency on the cortical surface of the participant reveals the retinotopic map in visual cortex as recorded with HD-DOT and (separately) with fMRI. [Adapted with permission from Eggebrecht *et al.*, NeuroImage **61**, 1120–1128 (2012). Copyright 2012 Elsevier.] (c) Retinotopic methods have also been utilized to quantitatively assess image quality of HD-DOT when using participant-specific anatomy as compared to when using atlas-derived anatomy for the light model. Atlas-based reconstructions produce similar image quality as participant-specific models as compared to fMRI-derived maps of brain function. [Adapted with permission from Ferradal *et al.*, NeuroImage **85**(1), 117–126 (2014). Copyright 2014 Elsevier.]

In a seminal study in 2005, Zhang and colleagues acquired data using simultaneously collected MRI and DOT. In response to five blocks with alternating fixation followed by a flashing black and white checkerboard pattern, results demonstrated bilateral visual cortex activations in both modalities.^192^ In 2007, an HD-DOT system using a closest source-detector separation distance of 13 mm was first reported. This HD-DOT system was shown to recover visual cortex activations in response to flickering checkerboard wedges displayed in each quadrant of the visual field in seven adult participants [Fig. 9(a)].^45^ The resultant images clearly delineated four quadrants of visual cortex activation intensities corresponding to visual stimuli in the upper and lower left and right visual fields. Several years later, light models utilizing realistic anatomy were used to reconstruct HD-DOT retinotopy data using subject-specific MRI-derived anatomy [Fig. 9(b)]. This study assessed visual cortex activity during separate HD-DOT and fMRI sessions in five healthy adults using a phase-encoded paradigm^174^ of rotating and flickering checkerboard wedges. Single-subject and group average data demonstrated that fMRI and HD-DOT retinotopic mapping boasted a high degree of correspondence in visual cortex:^47^ these methods recovered activations with an average localization error of 4.4 mm relative to subject-matched fMRI. Additional studies used retinotopy based paradigms to demonstrate atlas-based light models yield similar results to subject-specific anatomical models as compared with fMRI-based retinotopy mapping (with an average localization error of 6.6 mm relative to subject-matched fMRI) [Fig. 9(c)].^52^ Using atlas-based light models (such as with the MNI152 and Colin27 atlases) is advantageous for DOT imaging as it alleviates the necessity of acquiring a structural MRI image for each subject when there is adequate spatial agreement between the atlas and the subject/population.^87^

### B. Validation of HD-DOT with motor paradigms

**FIG. 10.**
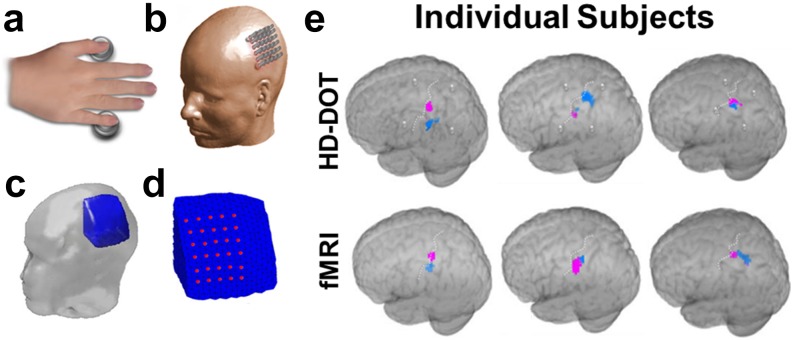
Validation of HD-DOT image quality using *in vivo* motor activations. (a) Vibrotactile stimulation was administered to the thumb and pinky finger. (b) A 4 cm grid of 30 source/detector fibers was placed over left motor cortex. (c) Location of the region used for light modeling within the participant’s head. (d) Finite element mesh model covering the field of view of interest (blue mesh) and the locations of the optical fibers (red dots) were defined individually for each subject. (e) Reconstructed thumb (pink) and pinky (blue) activations in motor cortex for each individual subject as assessed with HD-DOT or (separate) fMRI. [Reproduced with permission from Habermehl *et al.*, NeuroImage **59**, 3201–3211 (2012). Copyright 2012 Elsevier.]

**FIG. 11.**
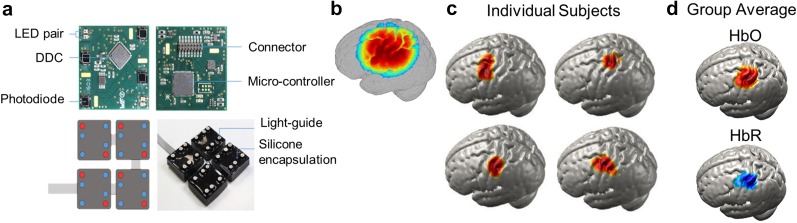
A wearable modular and fiberless on-head HD-DOT system design. (a) Each DOT module consists of four photodiode detectors (red) and two LED sources (red). Each detector is attached to the analog input of a charge-to-digital converter (DDC). (b) Normalized array sensitivity at 770 nm displayed on a cortical mesh of a single subject. (c) A motor task consisting of touching the thumb to finger was used to generate motor cortex activity in six adult participants (four shown). (d) Group average HD-DOT activations demonstrate oxygenated (HbO, red) and deoxygenated (HbR, blue) activations in motor cortex. [Adapted with permission from Chitnis *et al.*, Biomed. Opt. Express **7**, 4275–4288 (2016). Copyright 2016 OSA Publishing.]

The sensorimotor cortex provides an additional compelling location for simple and reliable validation of neuroimaging technology. The spatial organization of anatomical and functional areas along the motor cortex correspond to specific areas of the body, much like areas of visual cortex map to areas of the retina. Habermehl and colleagues examined HD-DOT activations within motor and somatomotor cortex in eight adults using vibrotactile stimulation of the thumb and pinky finger.^58^ Subject-specific modeling was used to tomographically map HbR motor activity onto the surface for each subject (Fig. 10). Distinct activations to thumb and pinky fingers were observed in five out of eight subjects using both HD-DOT and nonconcurrent fMRI. The localization error between HD-DOT and fMRI motor activations was estimated at approximately 10 mm. More recently, a fiberless HD-DOT imaging system demonstrated feasibility by reporting motor cortex activity observed in five adult subjects.^160^ Subjects were asked to touch the thumb to pointer finger of the dominant hand in 20 runs of 15–second blocks. Individual and group averaged oxygenated and deoxygenated activations were tomographically reconstructed on the surface (Fig. 11). Fiberless systems provide an exciting and compelling alternative to fiber-based HD-DOT. On-head optoelectronics provide a significant set of challenges and are discussed in more detail elsewhere.^165^

### C. Validation of HD-DOT with language paradigms

**FIG. 12.**
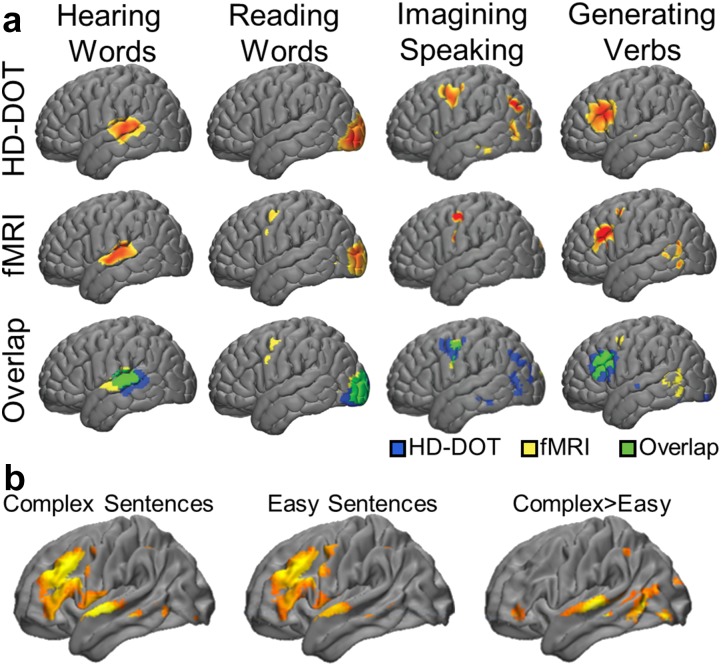
Validation of HD-DOT image quality across an extended field of view using language paradigms. (a) Language-related contrast-to-noise maps in a group of 5 adults. Task-based language paradigms included passive hearing words, silent reading of words presented on a screen, imagining speaking of the visually presented words, and covert generation of verbs associated with nouns presented on the screen. HD-DOT (top row) and fMRI (middle row) demonstrate a strong degree of overlap (bottom row) across the extended field of view. [Adapted with permission from Eggebrecht *et al.*, Nat. Photonics **8**, 448–454 (2014). Copyright 2014 Springer Nature Publishing.] (b) Complex sentences (containing object-relative clauses) and simple sentences (subject-relative clauses) were utilized to demonstrate strong left lateralized temporal and dorsolateral prefrontal activations when compared to noise-vocoded sentences. Brain areas associated with processing of semantic complexity of the sentences were found in left-lateralized regions of temporal, parietal, and dorsolateral prefrontal cortex. [Adapted with permission from Hassanpour *et al.*, NeuroImage **117**, 319–326 (2015). Copyright 2015 Elsevier.]

Brain areas associated with perception and generation of language are distributed throughout the cortex, including regions of temporal, parietal, and prefrontal cortex.^194–196^ Due to this extended and differentiated organization, studies validating the efficacy of HD-DOT for investigating language-based task paradigms have required a field of view that extended beyond that of primary sensory and motor regions. In order to validate the largest-to-date field of view HD-DOT system,^46^ the authors selected a hierarchical language paradigm first established in a seminal PET study that mapped the spatial topology of single word processing in the brain.^5^ During the hierarchical language paradigm, several different experimental probes of language function were utilized. In the first task, participants listened to a prerecorded list of single nouns. Each run consisted of six blocks within which nouns were presented one/s for 15 s followed by 15 s of silence (hearing words). Next, participants silently read a series of simple nouns displayed one at a time in the same block design on a screen (reading words). The third experimental run required participants to imagine speaking each word out loud (imagined speaking). Finally, participants were asked to silently generate associated verbs in response to each noun presented on screen (covert verb generation). Activation maps corresponding to each of these aspects of language were recorded during a HD-DOT imaging session followed by an fMRI session on a separate day. Strong agreement between HD-DOT and fMRI was apparent with robust contrast-to-noise activations in auditory cortex, visual cortex, superior temporal lobe, and dorsolateral prefrontal cortex apparent in both modalities [Fig. 12(a)]. This study highlighted the spatial correspondence of HD-DOT and fMRI throughout a spatially extended field of view that encompassed both sensory areas, known for exhibiting large signal to noise activations, as well as cognitive and association areas, known in the fMRI literature for exhibiting relatively smaller activation volumes and contrast levels. Group maps of activations detected with HD-DOT and fMRI showed strong concordance with responses to the simple perceptual tasks of hearing words and reading words in auditory and visual regions, respectively. The more cognitive tasks of imagined speaking the presented word or generating a novel verb revealed subtle differences beyond the agreement within motor and prefrontal areas, respectively, in the group maps between HD-DOT and fMRI. These differences are seen in cognitive regions within temporal, extrastriate, and parietal areas known to be associated with aspects of language processing that generally present with low SNR relative to sensory regions in response to these tasks. These differences are expected given the low number of subjects (N = 5) in this study and the level of intersession variability of cognitive language tasks for a given participant.

Additional studies of language processing with the HD-DOT large field of view system have investigated brain function underlying processing of syntactically complex and simple sentences. While syntactically complex and simple sentences both activated similar regions of cortex, including dorsolateral prefrontal cortex and auditory cortex, complex sentences elicited greater activations in primary auditory, ventrolateral prefrontal cortex, and temporal cortex than syntactically simple sentences [Fig. 12(b)].^33^ These results were largely consistent with prior fMRI and PET linguistic research and are suggestive of the validity and spatial specificity of task-based HD-DOT measured activations.

### D. Resting state functional connectivity HD-DOT

While task-based studies were particularly useful for validating event-elicited brain activations between HD-DOT and fMRI, task-free neuroimaging experimental methods have also been used to validate HD-DOT against fMRI. An increasingly common method in the fMRI literature is the use of resting state-functional MRI (rs-fMRI), a technique that can be used to assess functional connectivity within the brain in the absence of a stimulus. These resting state methods are ideal for situations in which a participant may be unable to engage in a traditional task-based block-design or event-related neuroimaging paradigm, such as infants or those who are asleep, anesthetized, or cognitively impaired. Functional connectivity can be inferred by assessing temporal correlations in low frequency fluctuations of the BOLD signal (in the range of 0.008–0.09 Hz).^161,197,198^ Importantly, rs-fMRI data can be used to identify spatially-distributed brain networks comprising regions of the brain known to be activated by task, including primary cortical regions such as visual and motor cortex, as well as higher order cortical areas supporting cognitive control, attention, and executive functions.^199–202^ The composition of these resting state networks has been well-characterized using fMRI in healthy adults and older pediatric populations^1–3,6,7,13,200,203–209^ and has also been increasingly utilized in infants.^210–217^

**FIG. 13.**
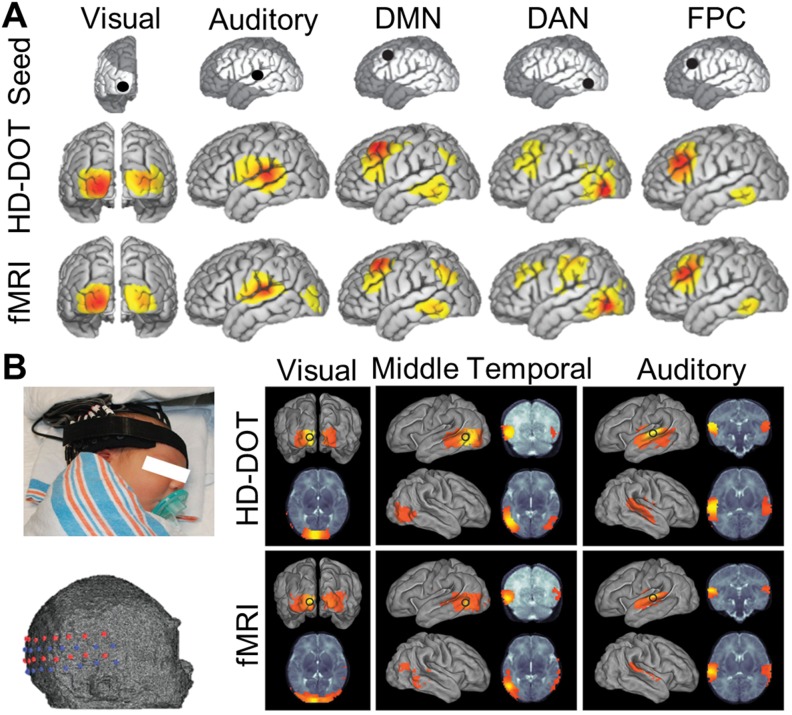
Validation of HD-DOT image quality using resting state functional connectivity and fMRI. (a) Functional connectivity maps generated from eight adult participants using seed regions of interest in the putative visual network, auditory network, default mode network (DMN), dorsal attention network (DAN), and frontoparietal control network (FPC). [Adapted with permission from Eggebrecht *et al.*, Nat. Photonics **8**, 448–454 (2014). Copyright 2014 Springer Nature Publishing.] (b) Functional connectivity maps generated from 14 term-born infants within the visual, middle temporal, and auditory cortex demonstrate strong spatial agreement between HD-DOT and separately recorded fMRI. [Adapted with permission from Ferradal *et al.*, Cereb. Cortex **26**, 1558–1568 (2016). Copyright 2016 Oxford University Press.]

Validation of resting state functional connectivity methods for HD-DOT, (i.e., functional connectivity DOT; fcDOT) were first demonstrated in healthy adults.^60^ This seminal paper established that fcDOT maps were reproducible in participants across days and that bilateral maps of strong correlations within (and not between) visual and motor regions were replicated in fMRI in those same participants. More recently, subject-specific light modeling and an expanded field of view broadened the reach of fcDOT methods to map not just sensory or motor networks, but also spatially distributed cognitive networks including cortical aspects of the dorsal attention network, fronto-parietal control network, and the default mode network [Fig. 13(a)].^46^ Group level analyses demonstrated similarities in the topology of these brain networks between fcDOT and subject-matched rs-fMRI. This type of analysis has also been extended to imaging functional connectivity in neonates [Fig. 13(b)]. Patterns of bilateral visual, middle temporal, and auditory cortex connectivity were observed using both HD-DOT and fMRI.^25^ Taken together, these validation studies suggest HD-DOT is capable of measuring both task-based activations and functional connectivity in human participants with comparable spatial specificity to that observed with fMRI.

## V. DOT APPLICATIONS IN HUMAN CLINICAL POPULATIONS

We will finish this review with a brief overview highlighting studies that have applied DOT and HD-DOT technology to clinical populations in environments beyond the reach of traditional methods such as fMRI. Imaging infants in the neonatal intensive care unit (NICU) with fMRI presents significant challenges for monitoring brain health. Neonates hospitalized for extended periods in the neonatal intensive care unit (NICU) may not be stable enough to move to an fMRI machine. For example, the most profoundly infirmed neonates may need mechanical ventilators, continuous positive airway pressure, or extracorporeal membrane oxygenation. Moving these infants for fMRI neuroimaging presents significant challenges for the health and safety of the patient. Thus, HD-DOT methods provide a compelling surrogate to fMRI and afford an opportunity to image cortical brain activity at the bedside. To-date, several studies have used DOT to assess brain activity and functional connectivity within preterm infants at a range of gestational ages recovering in the NICU. In this section, we highlight the use of optical methods with a focus on DOT in several case reports of infants with brain injuries including stroke, intraventricular hemorrhage (IVH), and hypoxic ischemic encephalopathy (HIE). Finally, we summarize combined EEG-DOT systems used to assess neonatal seizure activity and task-based activations in adult patients with epilepsy.

In the late 1990s, a groundbreaking paper reported the first DOT activations in an infant born extremely preterm (<27 weeks).^168^ These authors were able to measure motor activations while manually stimulating the left and right fingers. More recently, Hintz and colleagues similarly demonstrated motor cortex activity to passive arm movements in infants born moderately preterm (32–33 weeks gestational age).^218^ These early studies demonstrated the initial feasibility of DOT within the NICU.

**FIG. 14.**
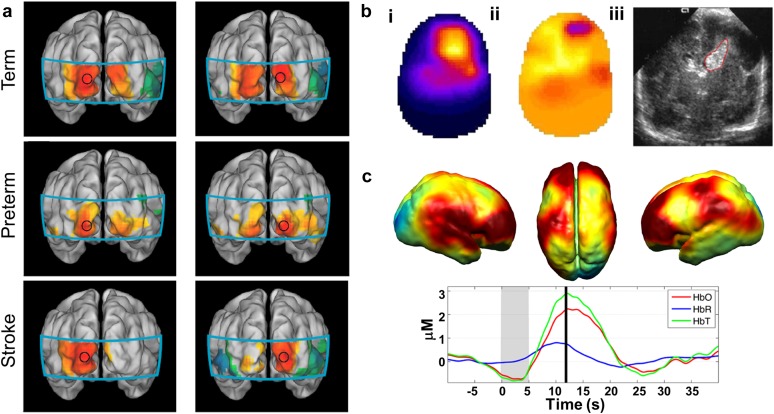
Application of DOT in studies of neonatal brain injury. (a) Maps of resting state functional connectivity using HD-DOT (fcDOT). A seed placed in left or right visual cortex reveals strong temporal correlations throughout contralateral visual cortex in term born neonates. Infants born preterm have weaker bilateral connectivity within the visual cortex. Bilateral visual network connectivity was completely absent in a preterm infant with a unilateral left occipital stroke. [Reproduced with permission from White *et al.*, NeuroImage **59**, 2529–2538 (2012). Copyright 2012 Elsevier.] (b) Whole brain reconstruction of time domain optical data acquired from a preterm neonate with left-sided intraventricular hemorrhage (IVH). Regional blood volume (i), regional oxygen saturation (ii), and cranial ultrasound demonstrate disruptions in oxygenation and hemoglobin concentration in the region near the hemorrhagic parenchymal infarct (iii). [Reproduced with permission from Austin *et al.*, NeuroImage **31**, 1426–1433 (2006). Copyright 2006 Elsevier.] (c) Whole brain DOT cortical reconstruction corresponding to the peak total hemoglobin concentrations following 4–6 s long EEG burst activity on a background of a burst suppressed EEG in an infant with grade III hypoxic ischemic encephalopathy (HIE). [Adapted with permission from Chalia *et al.*, Neurophotonics **3**, 031408 (2016). Copyright 2016 SPIE.]

While some studies rely on passive arm movements and tactile stimulation,^219^ other studies make use of passive brain activity while the infant is resting. Specifically, a single high-quality dataset can be acquired in minutes, and data can be collected from subjects swaddled, resting quietly, sleeping, and under anesthesia or morphine without requirement of task performance or attention to stimulus. White and colleagues acquired fcDOT on three term-born infants and four preterm-born infants using a HD-DOT cap covering left and right occipital lobes.^26^ Infants were imaged lying on their backs, with the weight of the fiber optics rested on the bed. One of the preterm infants exhibited a large left occipital hemorrhage, apparent on a T2-weighted MRI. Using fcDOT, bilateral functional connectivity maps were apparent within visual cortex in the healthy term-born infants [Fig. 14(a)]. This same pattern of bilateral visual cortex connectivity, although weaker, was also present in preterm-born subjects. However, bilateral visual cortex connectivity was absent in the preterm infant with left occipital hemorrhagic stroke.^26^ Similarly, researchers have recently demonstrated reduced interhemispheric connectivity in four infants following perinatal stroke as compared to four healthy infants.^220^

Neonatal brain injury, including the study of acute injury, such as IVH, is a particularly interesting application of DOT within the NICU. IVH typically occurs within the first 72 h following birth and is one of the leading forms of preterm brain injury.^221^ Austin and colleagues used a time-resolved DOT system known as MONSTIR (Multichannel Optoelectronic Near-infrared System for Time-resolved Image Reconstruction)^222^ to scan 14 preterm infants in the NICU, several of whom were diagnosed with IVH. DOT caps were constructed to cover the entire cortex, and each cap was custom-built to the head shape of the infant. Due to the long data acquisition time of this system, fcDOT was not performed. Instead, the authors generated mean photon flight times compared to a phantom reference volume. Using this analysis method, one preterm infant demonstrated increased regional blood volume and oxygen saturation [Fig. 14(b)] corresponding to IVH in the left hemisphere, visible on ultrasound.^223^ More recently, using a frequency domain DOT system (ISS Imagent™, Champaign, Illinois) recording at a 38.5 Hz frame rate with an irregular but high density cap configuration, researchers demonstrated decreased pulse rise time in ten preterm infants with IVH as compared to 20 preterm infants without IVH at various stages of recovery in the NICU up through term equivalent age.^157^ These results suggest imaging infants with DOT either during this acute period of brain injury or later during recovery in the NICU may provide insights into neural disruptions leading to subsequent neurodevelopmental impairment later in life.

Neonatal HIE has also been investigated using DOT. HIE occurs as a result of oxygen deprivation from fetal trauma either during gestation or during birth, and can result in long-term developmental complications including cerebral palsy, epilepsy, and sensory impairments.^224–227^ Infants with HIE provide a particularly compelling case for the use of DOT imaging, as the standard of care for infants with HIE is therapeutic hypothermia treatment for 72 h following birth. This therapeutic hypothermia treatment cools the body temperature of the infants, mitigating further brain damage resulting from the hypoxic event. However, the equipment used to cool the infant’s body temperature is not MRI compatible. Therefore, portable brain monitoring and imaging modalities such as EEG and DOT provide crucial clinical information about brain function during this treatment period. Chalia and colleagues used DOT to study hemodynamics associated with high-frequency bursting EEG activity, typically signifying pathological activity, in a group of term-born infants with HIE during the warming period following therapeutic hypothermia treatment in the NICU. Infants presented with seizures in the first 48 h of life and were scanned with combined EEG-DOT within seven days of birth. Across infants, oxygenated hemoglobin initially declined during the EEG bursts, and peaked 10–12 s after the bust onset.^27^ Though this study did not use a high density arrangement of measurements, this study provides a powerful example of the clinical application of concurrent EEG and DOT methods and illustrates the opportunity for advanced optical methods to inform clinical care [Fig. 14(c)].

**FIG. 15.**
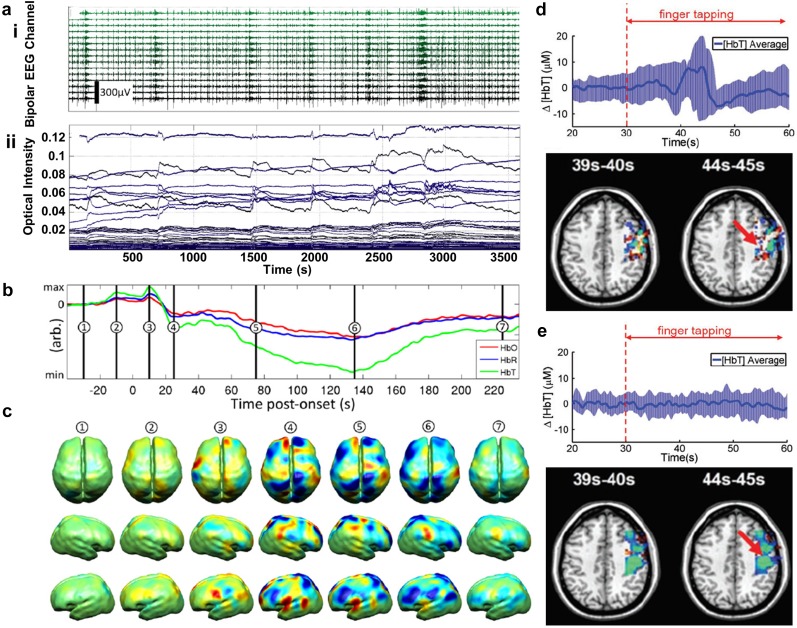
Application of DOT in studies of seizures in infants and adults. (a) Seizure activity in newborn infant with hypoxic ischemic encephalopathy (HIE). (i) Bipolar EEG channel timetraces demonstrate noticeable high frequency periods across most channels. (ii) DOT optical intensity changes in several source-detector pair amplitude increases mirror the high frequency periods observed in EEG. (b) Oxygenated (HbO), deoxygenated (HbR) and total hemoglobin (HbT) timetraces corresponding to spatially averaged brain activity following a seizure event at time point zero. (c) Maps of HbT at time points shown in B. [Reproduced with permission from Singh *et al.*, NeuroImage: Clin. **5**, 256–265 (2014). Copyright 2014 Elsevier.] (d) Finger tapping activity within the corresponding reconstructed activations in a healthy adult subject and (e) a patient with epilepsy. [Reproduced with permission from Dai *et al.*, J. Biophotonics **11**, e201600267 (2017). Copyright 2017 John Wiley and Sons.]

While seizure activity is typically measured using EEG/MEG, modalities sensitive to changes in electrical/magnetic fields, recently researchers have utilized DOT to investigate BOLD correlates of seizure activity. Seizures represent a major medical challenge for treating neonatal infants with HIE, and seizures are associated with poorer neurodevelopmental outcome. Singh and colleagues examined an infant with severe HIE during a 60 min period of passive rest following the warming period after 72 h of therapeutic hypothermia.^28^ The authors observed seven discrete periods of generalized whole-scalp EEG hyperactivity indicating seizure events [Fig. 15(a)]. Concurrent DOT imaging revealed HBT amplitude increases following each seizure event [Fig. 15(a)]. Averaging DOT activity across all channels following one of the seizure events revealed HbO, HbR, and HBT peak amplitude 15 s following seizure events [Fig. 15(b)]. The authors also observed spatial variation in the localization of activity prior to, during, and following the seizure events [Fig. 15(c)]. This study suggests the use of DOT in the clinic is a useful tool in addition to standard EEG bedside monitoring.

Limited prior work has imaged human adult clinical populations using DOT or HD-DOT. One prior study imaged three healthy adults and three adults diagnosed with temporal lobe epilepsy as a proof of concept.^158^ While seizure activity was not recorded, in this study, the authors demonstrate DOT activation differences in adults with and without epilepsy during a finger tapping task. Changes in HbT amplitude were observed in motor cortex of healthy adults [Fig. 15(d)], while adults with epilepsy showed no signs of hemodynamic response [Fig. 15(e)]. The authors suggest this lack of hemodynamic response in the motor cortex of epilepsy patients results from “the epileptic lesion existing in the brain of patients.” The authors note that the patients with epilepsy did not suffer from any clinical motor impairments which might otherwise explain their apparent lack of motor activity.^158^

Cumulatively, these papers illustrate some unique opportunities for applying advanced optical methods in the clinic and potentially in basic neuroscience. Future adult and infant studies would benefit from improved reliability and image quality afforded by utilizing whole-head HD-DOT imaging. For example, the silent and minimally constraining environment of HD-DOT may open the doors to neuroimaging studies on neural correlates of meditation and pharmacologically altered consciousness such as brought about via sedation, anesthesia, or psychoactive medications increasingly used to treat depression, post-traumatic stress disorder, or other conditions. Additionally, due to the lack of contraindications for implanted metal, HD-DOT may be used on studies involving participants with neural prosthetics such as deep brain stimulators and cochlear implants.^46^

## VI. CONCLUSIONS

In this review, we have focused on the physical principles underlying optical neuroimaging in humans, and the challenges of design and implementation of high density arrays. We have highlighted several studies that have demonstrated strong validation of the anatomical specificity and reliability of the technology. Finally, we summarized papers highlighting the unique potential for HD-DOT methods to profoundly impact clinical care.

Some limitations of HD-DOT should be discussed. The sensitivity of HD-DOT degrades with depth, as with all optical methods and validated imaging with HD-DOT beyond ∼15–20 mm from the surface has yet to be presented. The most robust strategy for overcoming that degradation is to increase the number of measurements at longer distances. However, as discussed above, as the source-detector separation increases linearly, the light level at the detector falls off exponentially. As such, significant advances in deeper imaging will most likely come about due to advances in detection and ADC technology with lower noise floors and wider dynamic ranges potentially along with advances in longer wavelength sources and detectors. Even with those potential advances, HD-DOT, unlike methods like fMRI, is limited to imaging the superficial cortex. Therefore, HD-DOT cannot access deep cortical structures such as the insula and operculum or deep subcortical brain structures such as the striatum, amygdala, hippocampus, or the thalamus.^46,47,146^ While this limitation is potentially a problem in mapping of functional connectivity networks, known functional connectivity networks have nodes in the superficial cortex.^1,46,228^ Furthermore, the functional connectivity structure of the brain has been shown to exhibit network-like properties, where a lesion within one area of the brain will often have effects that can be measured at spatially separated loci, often including those in superficial cortex.^229^ Even though HD-DOT is constrained to imaging brain activity in superficial cortical areas, disruptions throughout the functional brain network may be within reach.

Recent advances in HD-DOT systems make this modality a viable alternative to fMRI that can provide comparable spatial information about cerebral cortex activity and connectivity, with the added advantage of being portable (e.g., bedside data collection in populations that cannot be taken to a scanner). Multiple opportunities remain in the ongoing development of HD-DOT strategies for mapping human brain function. As discussed above, increasing the density of overlapping measurements has a direct positive impact on reconstructed image quality. One approach to increasing the density of overlapping measurements beyond current designs would be to lower the source-detector separation distances while maintaining the regular grid spacing. Though straight forward in design, this strategy leads to nontrivial challenges in cap design, cap fitting, source-detector encoding and decoding strategies, and the management of the hair of participants. An alternative approach to increasing the number of measurements afforded by a given arrangement of sources and detectors would be to use frequency domain or time domain strategies in a high density arrangement. The added phase- (or time-gate) based measurements compliment the intensity-based measurements and could provide further improvements in image resolution, localization accuracy, and quantification. Fiberless designs that place source, detector, and digitization components on the head have the potential to dramatically increase wearability and portability. All of these designs will also require further advancements in reliable and efficient anatomical co-registration methods, such as electromagnetic localization of the array and head fiducials or surface capture based on photometric strategies. Additionally, the increasing and large number of measurements required by HD-DOT arrays will require further developments in data fidelity assurance and motion-artifact detection and processing.
